# Establishing a List of Parameters and Their Empirical Calibration via Simulations of Some Experimental Graphene-Based Transistors

**DOI:** 10.3390/mi17101165

**Published:** 2026-10-06

**Authors:** Cristian Ravariu, Bianca Adiaconita, Eugen Chiriac, Marioara Avram

**Affiliations:** 1 Department of Electronic Devices Circuits and Architectures, National University of Science and Technology “Politehnica” Bucuresti, Str. Splaiul Independentei no. 313, 060042 Bucharest, Romania; 2 Department of Micro and Nano-Fluidics, National Institute for Research and Development in Microtechnologies-IMT, Str. Erou Iancu Nicolae no. 126A, 077190 Voluntari, Romania; bianca.adiaconita@imt.ro (B.A.); eugen.chiriac@imt.ro (E.C.); marioara.avram@imt.ro (M.A.)

**Keywords:** graphene, transistors, simulations, device parameters, static characteristics

## Abstract

In the actual microelectronics context, transistors with alternative semiconductor materials, using elements from the fourth group, other than silicon or germanium—for example, carbon—have become materials of extreme interest in overcoming certain limitations experienced by traditional MOSFETs. Therefore, graphene field-effect transistors (GFETs) offer the advantage of ambipolar conduction, providing static transfer characteristics dominated by ON current, I_ON_, both at positive gate bias for the electron accumulation channel and at negative gate bias for the hole accumulation channel. Between these two ON states, a Dirac point arises as a minimum current, representing the moment of compensation between electron and hole currents, which essentially defines the OFF current, I_OFF_. Due to this future development of GFET transistors, it is necessary to propose simulation techniques for optimizing these electronic devices. GFET simulations are present in the literature, but further calibrations are necessary, since graphene does not exist as a defined semiconductor material in the usual Technology Computer-Aided Design (TCAD) libraries. In addition, the properties of each graphene material differ from one experimental realization to another, depending on the technology applied by each author. This article starts with some experimental transfer characteristics measured for two types of fabricated GFET devices. Then, this study presents a few calibration techniques to simulate the graphene layer within GFET, aiming to fit the experimental transfer characteristics with the simulated curves while keeping some optimized technological sizes. The final goal of this article is to establish a list of GFET device parameters linked to the material properties and to estimate them through successive simulations, using empirical calibrations, for each type of experimental graphene material. This algorithm is not a validated procedure; instead, it indicates a set of sensitive GFET parameters, which allow for the adjustment of the shape and order of magnitude of the simulated current with the experimental one for given static characteristics.

## 1. Introduction

The intensive investigation of field-effect transistors with graphene materials instead of silicon was stimulated after 2010, when a Nobel Prize in Physics was awarded on this topic. Thus, in the 16 years that followed, MOSFET transistors on silicon reached the size limits of scaling down, up to 1 nm, in perfect agreement with the predictions of the famous Moore’s law, which was enunciated 50 years ago. Silicon electronics are nearing their physical limits, driving researchers to find reliable alternative materials [1]. In this context, two-dimensional materials, particularly graphene, become serious candidates. Firstly, graphene oxide was widely explored for field-effect transistors (FETs) due to its high electrical conductivity [2,3,4] and for biosensors due to carbon’s biocompatibility with organic matter [5], as essential features for label-free sensors capable of detecting trace amounts of chemical and environmental analytes [6,7,8].

Focused investigations of graphene field-effect transistors (GFETs) instead of silicon metal–oxide semiconductor field-effect transistors (MOSFETs) were spurred by the special properties of graphene in relation to electronics. With electron and hole mobility over 10 times higher than in silicon and a bandgap, E_g_, much smaller than that of traditional semiconductors in the fourth group, graphene gathers and amplifies all the useful properties of known semiconductors [9,10,11].

Organic semiconductors offer ambipolar conduction [11,12], similar to graphene, but with much lower mobility, between 10^−6^ ÷ 10 cm^2^/Vs [13,14]. Moreover, Si has a bandgap, E_g_, of 1.1 eV, while mono-crystalline carbon, as a diamond, has an E_g_ value that is much higher, i.e., approximately 5.45 eV, thus greatly limiting their electrical conductivity [15].

On the other hand, GFETs are also being researched to keep pace with the latest generation of MOSFETs. But perfect tuning among them is not yet possible. Down-scaled MOSFET in silicon offers an excellent I_ON_/I_OFF_ ratio of 10^6^, a low enough threshold voltage till of 0.3 V [16], a moderate ON current of a few micro-amperes [16], limited cutoff frequency [17], and a limited sub-threshold slope over 70 mV/dec [18], associated only with inversion channel, under unipolar conduction constraints. However, graphene-integrated sheets within MoS_2_ field-effect transistor revealed a sub-60 mV/dec slope that overcomes the ideal limit of MOSFETs [19]. Graphene exhibits a remarkable combination of properties: ambipolar charge transport with intrinsic carrier mobility exceeding 10^4^ cm^2^ V^−1^ s^−1^ on SiO_2_ substrates [20] and 10^5^ cm^2^ V^−1^ s^−1^ for suspended layers [21,22], a high surface-to-volume ratio that renders every atom accessible to the local chemical environment, and an almost-zero-bandgap energy-band structure, characterized by a tunable charge neutrality point—the Dirac point—whose voltage shift constitutes the primary sensing signal in graphene-based FETs (GFETs) [6,7,22,23,24,25].

Looking at the literature, it can be observed that there is a poor strategy for GFET simulation, as an electronic component itself. Few recent papers were rather focused on the simulation of the graphene atomistic structure or material properties [26,27].

The second type of GFET simulations, also rarely disseminated in the literature, envisage the simulation of integrated circuits based on GFET, establishing mathematical models [28], Spice-like compact models [29], or an equivalent circuit with passive components [30]. For instance, in [30], a circuit analysis with a GFET frequency double, whose fabrication was reported in [31], was presented, following a compact model for simulations of circuits with GFET to assess the device’s performance parameters.

An interesting paper was presented in 2021 about 2D GFET modeling and simulation, ultimately integrating some equations for Spice-like models in general software, like MATLAB, or behavioral circuit software, like Verilog [32]. This paper detailed different positions of electrodes, like top/back-gated graphene transistors, materials for substrate (quartz/glass, silicon, and silicon carbide), and the growing technology of graphene (CVD on Si, CVD on SiC, and exfoliation effects). For work regimes (like saturation and triode regime), they extended some Spice models previously reported [33]. Not only for GFETs but also for most TFT transistors, the main models were borrowed from MOSFET [34] or pseudo-MOS transistors [35,36] and expanded in terms of the meaning of the parameters, serving especially to the circuit for the displays’ simulation [37]. While the TFT, as a device with various layers of materials, has many publications on its simulation (e.g., [38,39]), including examples in the 2D simulator manual from Silvaco [40], the GFET has only a few incipient works [41,42,43].

The 2017 article proposed a starting structure based on a GFET transistor, where graphene was modeled as a 5 nm thick deposited polysilicon layer, in which the carrier mobility was redefined to 10,000 cm^2^/Vs, approximating E_g_ = 0 for pristine and the electron saturation velocity was set to 4 × 10^7^ cm/s [41]. Their simulated transfer curves, I_D_-V_G_, at V_DS_ = 0.1 V and V_DS_ = 0.2 V, with the gate voltage swept from −3.5 V to +3.5 V, exhibited a Dirac point at V_G_ = −0.8 V, with an I_ON_ of 1.8 pA and an I_OFF_ of 0.4 pA [41].

A more complex device was simulated in 2022, starting with an Athena-based simulation of the fabrication process, followed by electrical simulation in a 2D device simulator of a 22 nm MOSFET featuring a 1 nm bilayer graphene sheet and 3 nm titanium dioxide (TiO_2_) as gate oxide, aimed at MOSFET-GFET co-integration technology GFETs [42]. The Shockley–Read–Hall (SRH) model was adopted as a physical model for the recombination mechanism and Lombardi CVT model for carrier mobility in graphene. Additionally, specific material parameters in graphene film are defined: the carrier mobility of 10,000 cm^2^/Vs, an approximated E_g_ of 0.55 eV [42], and electron/hole concentrations determined via N_C_, N_V_ effective energetic density of states. Numerical simulations were performed using Newton and Gummel methods. Although sub-1 V drain and gate voltages were applied the simulated static characteristics rather look like those of a MOSFET: a low threshold voltage V_T_ = 0.201 V, a high conduction current I_ON_ = 4228 mA/μm, but an almost zero leakage current I_OFF_ < 34 nA/μm for any V_G_ < V_T_, without a Dirac point, while a sub-threshold swing of 71 mV/dec was similar to that of a MOSFET [42].

In 2023, some authors were focused on the tunneling probability, symmetric band structure, low effective mass and general material characteristics of 2D atomic layers to simulate Graphene Tunnel Field Effect Transistor (GTFET). They modified the user-defined TCAD syntax for SiC to adapt it to the graphene layer [43].

None of the aforementioned studies demonstrated correlation between the simulated and experimental characteristics. Therefore, in this paper, the following objectives and aims of research should be addressed: (i) to simulate a standalone GFET using TCAD tools, incorporating various layers, electrical stimuli and current-voltage monitoring, excluding any GFET circuit monitoring at this stage; (ii) to present alternative solutions able to simulate graphene-based layers by 2D device simulator tool; (iii) to create a particular input TCAD model able to simulate a general GFET; (iv) to particularize the input data so that the simulated structure closely matches two types of experimental GFETs; (v) to capture the ambipolar conduction through graphene via simulations for both generalized and particular GFET structure; (vi) to propose a list of material parameters—grounded in both physics of semiconductors and the TCAD tool—whose values significantly influence the GFET’s transfer characteristics; (vii) to propose an empirical calibration methodology able to correlate the simulated with experimental static characteristics; and (viii) to define some error functions and to target their minimization in order to empirically calibrate, by trials at this stage, the parameter list as the final result of this paper.

## 2. Materials and Simulation Methods

### 2.1. What Materials and Experimental Devices Are Simulated

A major debate still divides the field: what is the ideal structural form of graphene for FET applications? Traditionally, Single-Layer Graphene (SLG) has been favored due to its excellent carrier mobility; however, it suffers from the disadvantage of complex, defect-prone transfer processes [44]. Recently, research focus has shifted toward Graphene Nano-Walls (GNW) and Nano-Crystalline Graphene (NCG), because they can be grown directly onto Si substrate, entirely bypassing the need for graphene sheets transfer. SLG material, also known as pristine graphene, has semimetal behavior (E_g_ ≈ 0) and a compact 2D honeycomb carbon structure of one atomic layer thickness. Nevertheless, it requires an expensive technological transfer from an initial copper growth substrate to a Si substrate, a transfer that jeopardizes the mono-atomic layer integrity. Therefore, SLG material was excluded from the scope of this study at this stage.

**Figure 1:**
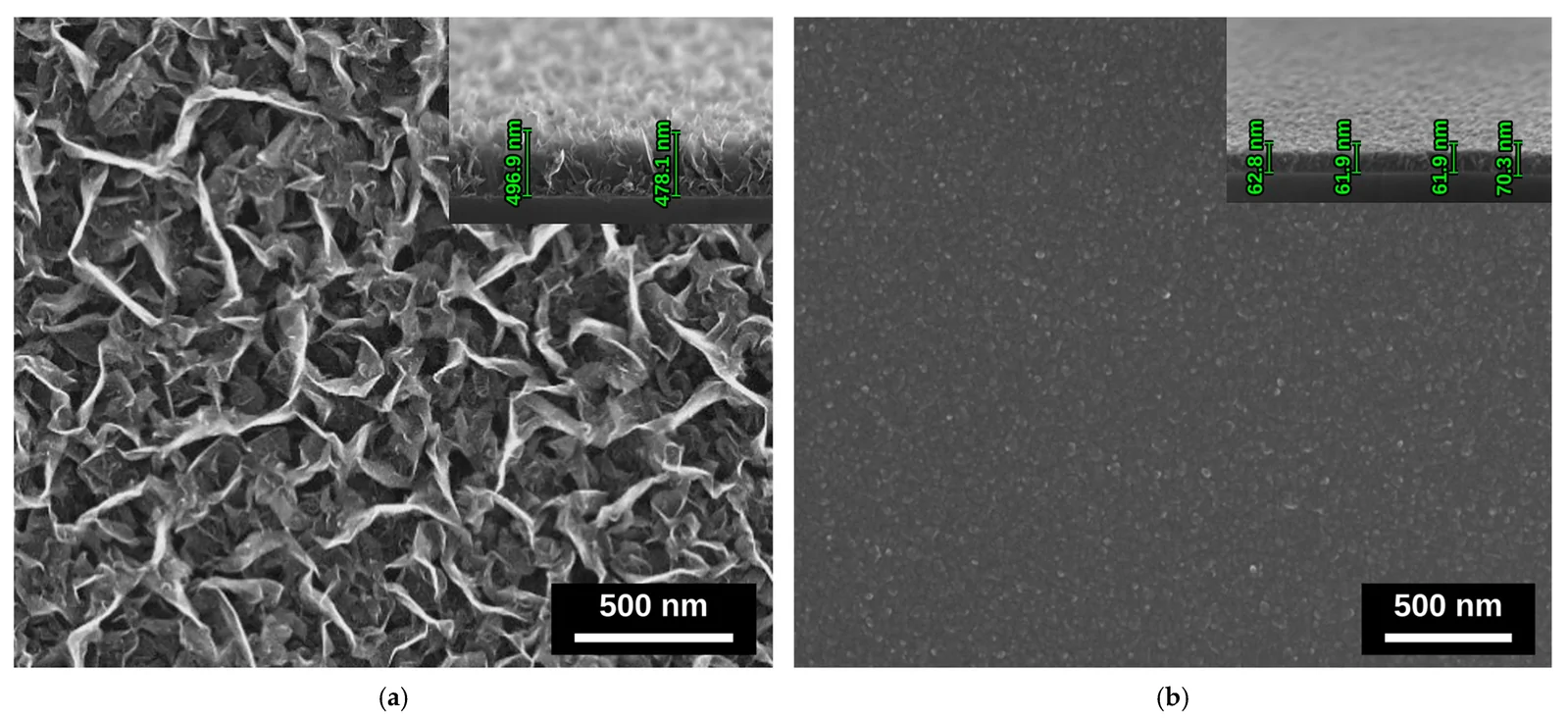
Detailed morphology of two graphene materials synthesized in our laboratories, analyzed by SEM, where 500 nm is a scale bar for: (**a**) GNW; (**b**) NCG.

On the other hand, the other two graphene-based materials were synthesized and deposited in our laboratories [44] to fabricate GFETs: (i) GNW, which exhibit semiconducting behavior with a small bandgap (E_g_ < 100 meV), where the carbon planes form nanodomains grown perpendicularly to the substrate surface, resembling a vertically aligned “forest” of carbon sheets; (ii) NCG, a representative of nanocrystalline graphite, with a semiconductor behavior, too (E_g_ < 100 meV), where the non-uniform sheets of carbon tend to possess an average orientation parallel to the substrate surface. All these graphene-based materials keep the primary graphene’s properties, while their structural similarities, associated with their affiliation to the graphene class, were demonstrated elsewhere by Raman spectra analysis [45]. The difference among them is rather given by their surface aspect, precursors and technology of deposition, which are extensively discussed in [45,46]. The surface morphology of the two synthesized GNW and NCG materials, directly grown on Si wafer, are emphasized here through the SEM images presented in Figure 1a,b.

These two types of materials can be deposited directly onto Si substrate and no longer require transfer techniques. Furthermore, all these graphene-based materials are accepted as undoped semiconductors, capable of generating GFET transistors with a detailed technology description in [45,46]. To distinguish between them, the following notations will be used throughout the paper: GNW-FET and NCG-FET represent those FET transistors with GNW and NCG films, respectively.

After the experimental tests of different graphene thicknesses, different gate insulator thicknesses and different graphene lengths/widths [45], the most successful structure achieved at minimum technological cost and generated a common geometry [46], whose features are considered as input parameters for GFET simulations. Based on the experiments conducted in 2023 [45] and the subsequent optimization of raw material compositions in 2025 [46], the thickness of the simulated graphene films was chosen to be 200 nm and the thickness of the Al_2_O_3_ film to be 10 nm as optimum values. These sizes are selected because the actual simulations must serve for the future optimized GFET device applications. But the technological methodology of GFET fabrication and their measurement techniques are the same as those depicted in [45,46].

The next main target of this paper is to find a list of device and material parameters that can adjust and calibrate the drain current via empirical trials and error minimization. The second goal is to group these parameters and to extract them. The last aim is to estimate by simulation to what extent the parameters influence the deviation of the transfer characteristics, emphasizing the ambipolar conduction in graphene.

### 2.2. What Transfer Characteristics of the Experimental Devices Are Considered

**Figure 2:**
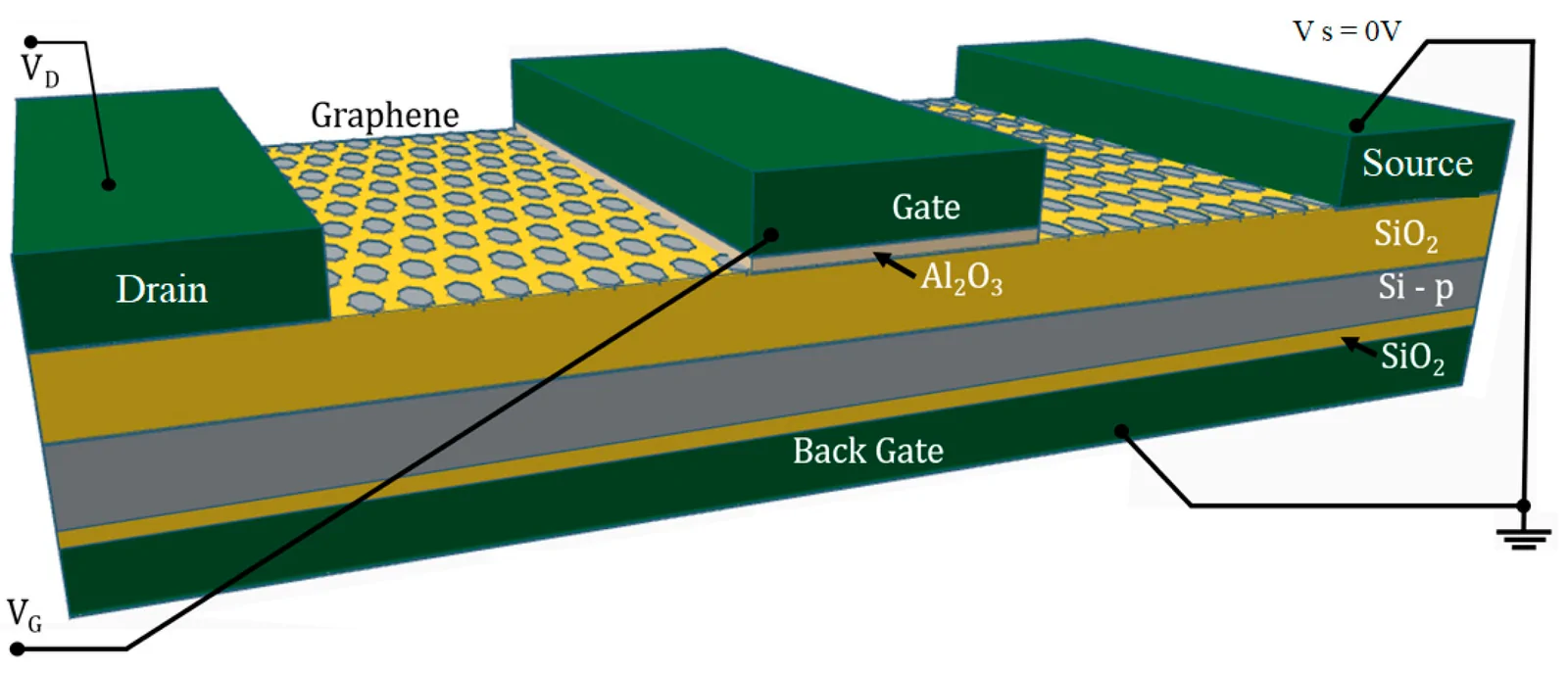
Basic structure and biased electrodes—a common structure for experimental and simulated GFETs, redrawn from [45], with MDPI open-access permission.

**Figure 3:**
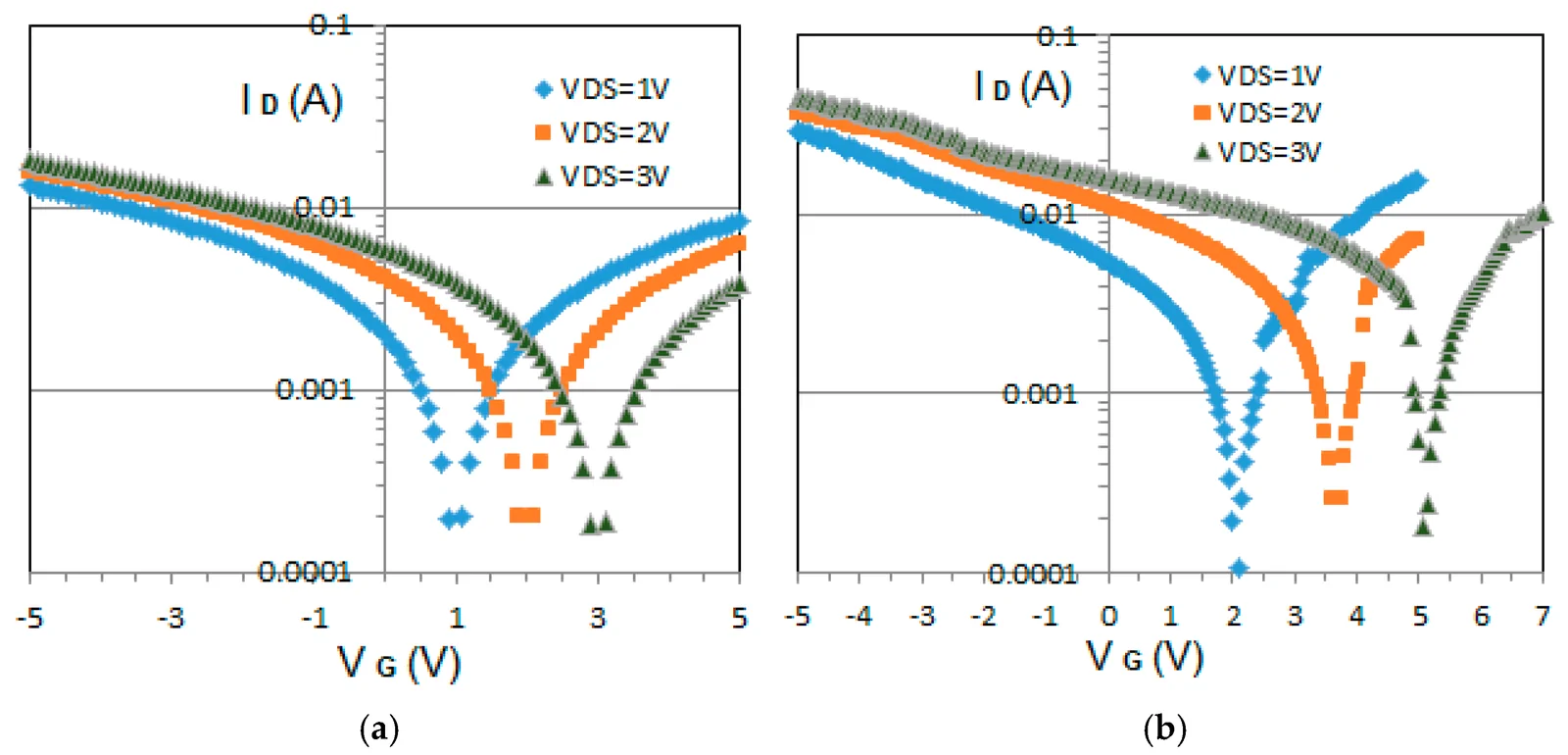
Measured transfer characteristics of different graphene-based transistors: (**a**) GNW-FET; (**b**) NCG-FET.

All simulated GFETs possess the same aspect as the experimental device, indicated in Figure 2. The following experimental transfer characteristics, I_D_-V_G_, were measured at V_DS_ = 1 V, 2 V and 3 V, respecting the measurement protocol previously reported [45,46] and taking care that the gate current is a leakage one, sub-50 μA. The measured curves from Figure 3 become the target of simulations for NCG-FET and GNW-FET.

From the experimental data (Figure 3), the I_D_-V_G_ measurement points were individually extracted at a voltage step of 100 mV and tabulated for subsequent numerical processing.

Although ambipolar conduction exists at any V_G_ value in the experiments, the maximum drain current for all measured GFET curves is encountered only at V_G_ < 0, while the maximum current along one curve occurs at V_G_ = −5 V; Figure 3.

### 2.3. What Input Parameters Are Used to Initiate the GFET Simulations—Set-I

For all GFETs there is no doping process executed in graphene. But the electrons and holes exist in graphene film, and their concentration depends on their density of energetic states in the conduction band (N_C_) and valence band (N_V_) and intrinsic concentration (n_i_). The energetic densities N_C_, N_V_ are related by n_i_ and E_g_ values [47]:

(1) $$n_{i}=\sqrt{N_{C}N_{V}}\cdot \exp\left(-\frac{E_{g}}{2\mathrm{kT}}\right)$$

The π bands of carbon materials are close to electron–hole symmetric distribution, so that a hole-to-electron effective mass ratio in the unity vicinity is expected. Consequently, the maximum range for Nv/Nc ratio will be searched between extreme limits like (10^−1^, 10^+1^).

(2) $$\mathrm{If}\mathrm{Nc}=\mathrm{Nv}\Leftrightarrow N_{C}=N_{V}=n_{i}\cdot \exp\left(\frac{E_{g}}{2\mathrm{kT}}\right)$$

Initially, considering N_C_ = N_V_ in (1) and assuming for all graphene types an average E_g_ = 60 meV [46,47] and n_i_ = 5 × 10^16^ cm^−3^ [45,46,47], according to (2) results, the input values for initial simulations are: N_C_ = 1.6 × 10^17^ cm^−3^ and N_V_ = 1.6 × 10^17^ cm^−3^. Initially, the energetic affinity for graphene, Ea, is considered 4.5 eV as for carbon materials [40], and the source and drain metallization made by Au/Cr layers exhibits an electron work function ranging from 5.1 eV specific to pure gold, up to 4.5 eV for pure Cr. The literature claims high values of the carrier’s mobility in graphene compared to traditional MOSFETs, ranging from 5000 cm^2^/Vs [48] to 10,000 cm^2^/Vs [21] and up to 100,000 cm^2^/Vs [21,49], while the electron saturation velocity is 4 × 10^7^ cm/s [42,50]. In the initial simulation, the electron and hole mobilities are estimated using average values: μ_n_ = 9000 cm^2^/Vs and μ_p_ = 10,000 cm^2^/Vs for electrons and holes, respectively, for all graphene types, according to [44,45,46]. In the next sections, the subsequent notations are introduced: Q_it_ is the interface flat-band charge [47], expressed as the net electron charge per cm^2^ at the interface between the graphene film and the Al_2_O_3_ gate insulator [14,50], N_tA_ and N_tD_ are the densities of acceptor and donor traps, respectively, that are initially neglected [50,51], V_DS_ is the drain-source voltage, V_G_ is the gate voltage and I_D_ is the drain current.

The following constructive and geometric parameters will rest constant during the next iterations and simulations for all types of GFETs: graphene thickness of 200 nm, placed onto SiO_2_/Si substrate, SiO_2_ thickness of 100 nm, Si thickness reduced to 100 nm, graphene length of 100 μm and graphene width of 100 μm, upper gate oxide Al_2_O_3_ of 10 nm, source–drain–gate contacts made by 200 nm metal whose work function was manually defined in contact statement, dielectric relative permittivity of graphene takes different values (2.4 in [42]; 3.3 in [43]), but a default value for C-related materials was maintained in this work k = 5.5 [40], and Si-substrate doping concentration of N_A-Si_ = 10^15^ cm^−3^ that is p-type Boron-doped, similar to experiments depicted in [45,46].

Because all the experimental currents are represented at logarithmic scale [45], the error function at the intermediary step i, Er − i that refers to the difference between the simulated and measured drain current, is computed using logarithmic values of the drain current:

(3) $$\mathrm{Er}-i=\left|\frac{|\mathrm{lg}I_{D,\exp,i}|-|\mathrm{lg}I_{D,\mathrm{sim},i}|\;}{\mathrm{lg}I_{D,\exp,i}}\right|$$

where I_D,exp,i_ and I_D,sim,i_ denote the experimental and simulated drain current, respectively, at the i-th increment within the gate voltage range of −5 V < V_G_ < +5 V. The global error function, Er − g, computes the sum of all individual Er − i errors along the entire simulated versus measured I_D_-V_G_ curve for $i=\bar{1,N}$ and then divides it by N, where N is the total number of increments:

(4) $$\mathrm{Er}-g=\frac{\sum_{i=1}^{N}\mathrm{Er}-i}{N}\cdot 100\;[\%]$$

The target of the subsequent simulations are: (i) to discover what device parameters have to be considered; (ii) to group the device parameters in the next analysis; (iii) to find similar simulated static characteristics in shape and range as experimental curves; (iv) to calibrate the device parameters by empirical methods, and (v) to minimize the global error, as the calibration criterion.

Only at the final stage, will a second error function, Root Mean Square Error (RMSE), be considered to monitor the experimental versus simulated currents, at V_DS,j_ = 1 V, 2 V and 3 V, and it will be used only in the Discussion section, according to the following expression:

(5) $$\mathrm{RMSE}=\sqrt{\frac{{\sum_{i=1}^{N}\left(I_{D,\exp,i}-I_{D,\mathrm{sim},i}\right)}^{2}}{N}}\cdot 100\;[\%]$$

where j corresponds to V_DS,j_ that can be 1 V or 2 V or 3 V, $j=\bar{1,3}$, and i is the increment for gate voltage increasing as in Equation (4). In all error functions, the drain current was expressed in Ampere.

### 2.4. Simulation Set-Up

The simulated structure starts from the definition of a rectangular domain on the xy-axes, inside which the material zones are defined, keeping the previous dimensions. A two-dimensional device simulator is used (e.g., Atlas from Silvaco 2017), for which the default width on the z-axis is 1 μm. Due to this 2D simulation convention, throughout this article, simulated drain currents I_D,sim_ will be expressed in A/μm (i.e., in A/1 μm). Conversely, experimental drain currents I_D,exp_ will be expressed in A (that means A/100 μm), representing the total current across the actual 100 μm width of the graphene layer. Therefore, to compare a simulated current with an experimental one, the current I_D,sim_ will need to be multiplied by 100 to be converted from A/1 μm to A/100 μm.

**Figure 4:**
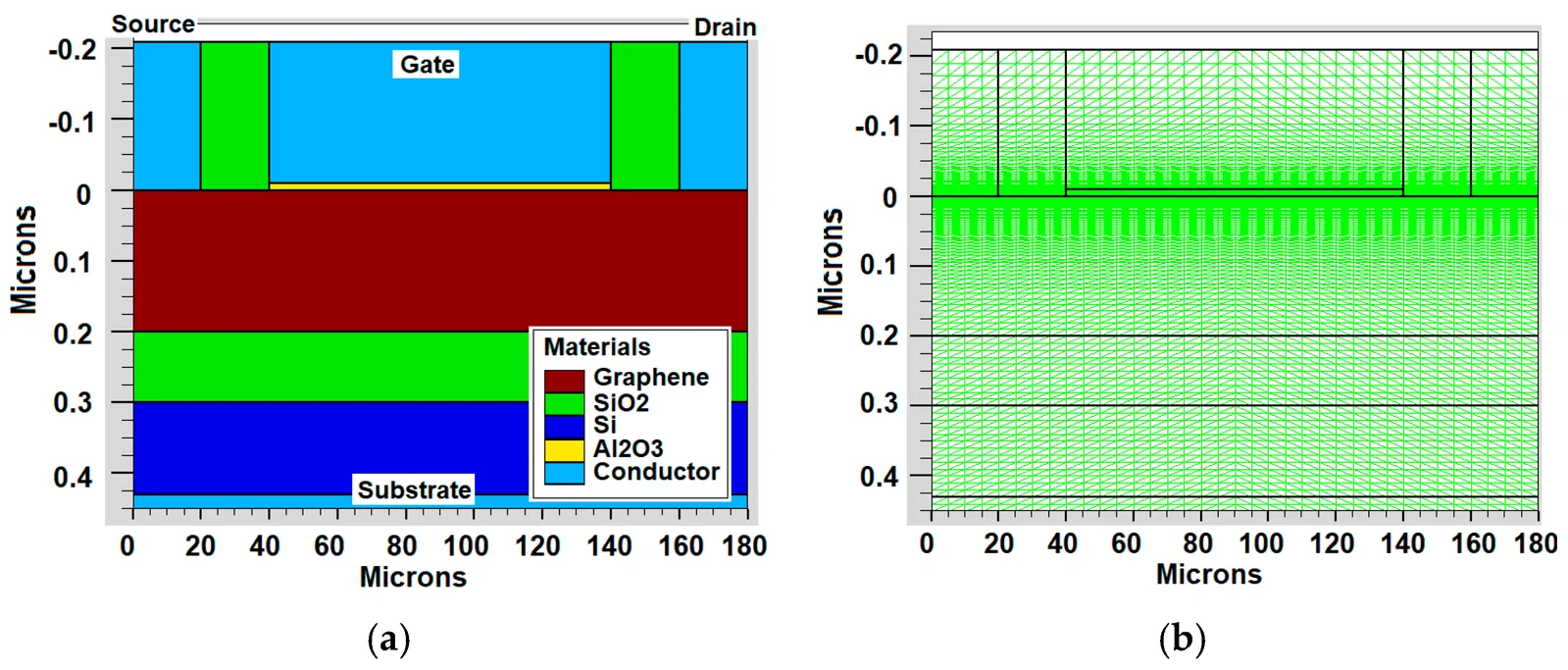
(**a**) The simulated GFET geometry and layer dimensions; (**b**) selected mesh.

Figure 4a presents the cross-sectional view of the simulated device that borrows the experimental architecture depicted in [45,46] and Figure 2, updated with previous optimized sizes. Figure 4b indicates the adopted discretization mesh. In this 2D device simulator, defining an appropriate mesh for a field-effect transistor (FET) is the critical initial step in device simulation. The goal is to create a fine grid in regions where physical properties exhibit rapid variations—such as Al_2_O_3_, graphene film, the channel region, and the interfaces of source/drain metals on graphene, in the case of our GFET. Concurrently, a coarser grid is maintained underneath the oxide and Si substrate to minimize the simulation time.

The technique of reducing the substrate thickness is often used in 2D device simulations to reduce the total node count and improve the aspect factor. In this configuration, the SiO_2_ and Si films underneath the graphene lack a functional role, as long as no electrical stimuli are applied to the back gate. Consequently, in the actual GFET, the SiO_2_ and Si film thickness were reduced to 100 nm, as illustrated in Figure 4a,b.

Since graphene material does not exist in the simulator’s material library it is defined as user-defined semiconductor, composed of carbon atoms, similar to the existing diamond material [51,52]. Models and methods are selected to ensure ohmic contacts for the source and drain metals to include an accurate mobility model to both temperature and electric field, such as the CVT Lombardi initial model [40] and a standard Shockley–Read–Hall (SRH) recombination model for two-carrier conduction. Simulations are conducted at a baseline temperature of 300 K and interface charge (Q_it_) characterized by initially a low value of 3 × 10^10^ e/cm^2^ [47], while N_tA_, N_tD_ parameters are initially neglected. To solve the semiconductor transport equations, a Newton numerical algorithm with a maximum of 40 iterations is employed. The reunion of the previous parameters forms the *Set-I* of parameters, at the I-st iteration, which are initially common for generic GFET, GNW-FET, and NCG-FET.

## 3. Simulations of GFETs and Parameters Calibration

### 3.1. GFETs Simulation at the I-st Iteration—Initialization

In this section the simulations start with a generic GFET structure that initially combines features of both GNW-FET and NCG-FET, and they share common initial parameters. Due to the symmetry of the source and drain electrodes, the potential difference between them is the governing factor, rather than their physical ordering. So, the source-drain or drain-source voltage of +2 V is applied in simulations and the notation V_DS_ = +2 V is used. The primary goal of this paragraph is to bring the simulated transfer characteristics, or at least its envelope, as close as possible to the measured characteristic at V_G_ = −5 V and at V_G_ = +5 V, where the currents are the strongest.

**Figure 5:**
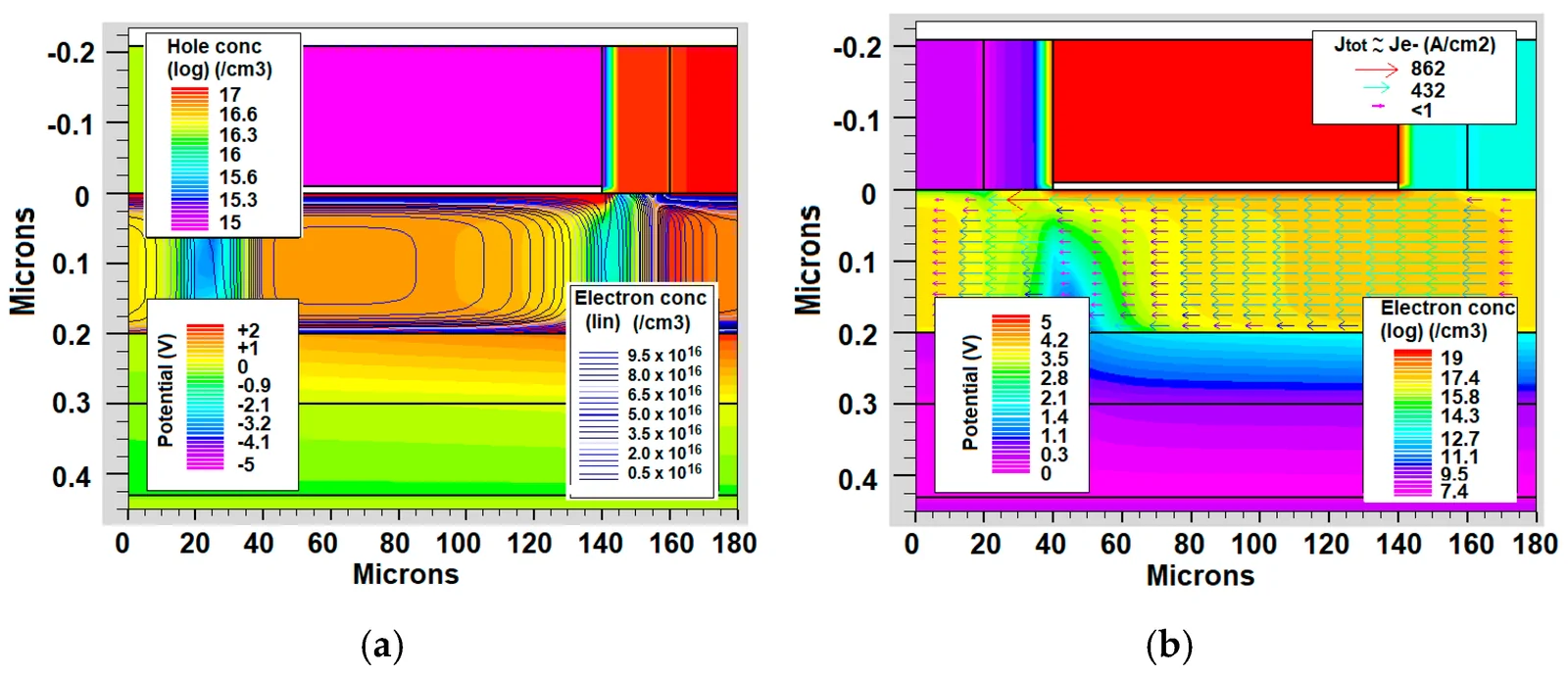
Potential is represented in all materials except graphene as colored contours; both electrons and holes reach comparable concentrations for two GFET biases: (**a**) V_G_ = −5 V and V_DS_ = +2 V; (**b**) V_G_ = +5 V and V_SD_ = +2 V.

The ambipolar operation of GFET is verified in Figure 5. To prove the electrodes biasing at V_G_ < 0 and at V_G_ > 0, the potential is represented as colored contours in all materials except graphene. The carrier concentrations are represented within the graphene film. Since the transistor is biased at V_G_ = −5 V and V_DS_ = +2 V, a hole accumulation channel is induced at the top graphene surface, beneath the gate, while the graphene film is depleted of electrons from the top to the bottom of the film. Indeed, Figure 5a shows that the maximum hole concentration at the graphene surface is *p* of 10^17^ cm^−3^, which is slightly higher than the maximum bulk electron concentration n of 9.5 × 10^16^ cm^−3^. In the same figure, rainbow-colored contours are used for the hole concentration and blue-colored lines for the electron concentration within the graphene film. This fact proves a conduction based on both electrons and holes, i.e., ambipolar transport, which is consistent with behavior reported in various other devices [53,54,55,56,57]. In this bias regime, the holes transport is concentrated at the upper Al_2_O_3_/graphene interface as an accumulation current, while the electron current is distributed through the bottom half of graphene, as a bulk current. In contrast, at a positive gate voltage, V_G_ = +5 V, an electron accumulation channel is induced at the graphene surface, which dominates the conduction, besides to a poorer hole current through the bulk; Figure 5b. The proof of the dominant electron current is the total current density representation by colored vectors, J_tot_, in Figure 5b, which exhibits its highest values at the graphene surface, matching the region of maximum electron concentration.

**Figure 6:**
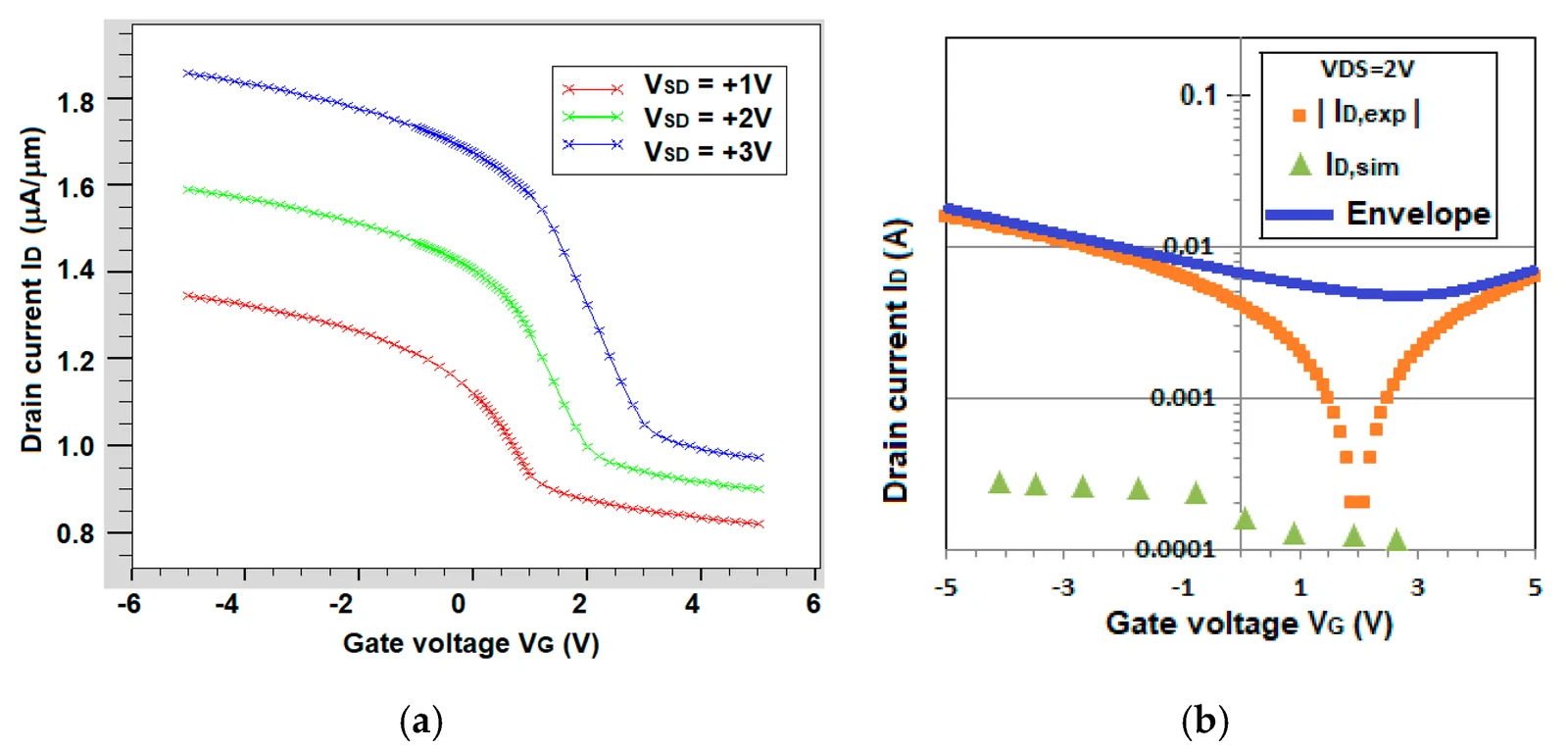
(**a**) The transfer characteristics simulated at V_DS_ = +1 V, +2 V and +3 V for a generic GFET; (**b**) comparison between simulated and experimental lgI_D_-V_G_ points, at V_DS_ = 2 V, using the experimental GNW-FET and all currents expressed in A.

At the I-st iteration, the following family of transfer characteristics (I_D_-V_G_) is simulated, adopting all the previous GFET material and device characteristics; Figure 6a.

As an observation, only the strong accumulation regimes (i.e., either at V_G_ = −5.... −1 V or at V_G_ = +1....+5 V) are captured by the simulations, as almost constant current regimes, while the minimum Dirac point does not appear in the simulations in the vicinity of V_G_ = 0 V; Figure 6a. The simulated current is still much smaller than the measured one; Figure 6b. The simulations reveal a transition from a hole accumulation current at V_G_ < 0 to an electron accumulation current at V_G_ > 0. We will further refer to this behavior as a fit only on the envelope of the simulated curves with the experimental ones (see the envelope in Figure 6b).

Obviously, NCG-FET, with hundreds times higher current, Figure 3b, still provides higher errors than the simulated curves from Figure 6a.

The subsequent calibration iterations aim to achieve three primary objectives: (i) increasing the drain current by approximately 100 times for both GFETs, (ii) finding those parameters that capture real effects as many as possible in the simulations, and (iii) performing a comprehensive curve-fitting procedure that first aligns the overall characteristic envelope and subsequently targets the Dirac point region, where the drain current reaches its minimum.

In order to raise the drain current range, at least on envelope, in the II-nd iteration, the simulations will be guided by energy considerations; the band gap may deviate from the initially predicted value of E_g_ = 60 meV in graphene, while the harmonization of the electron affinity in graphene Ea with the electron affinity in the source and drain metal contacts, E_S-D_, can present decisive effects on the conduction currents, avoiding non-ohmic contacts [58].

In subsequent iterations, the increase in the electron/hole currents in graphene film will be operated from the perspective of charge carrier distribution by tuning the effective energetic density of states N_C_/N_V_, refining the mobility values μ_n_/μ_p_ by accurate models for mobility, and accounting for the defects density distribution.

### 3.2. GFET Simulation at the II-nd Iteration—Energy Considerations

**Figure 7:**
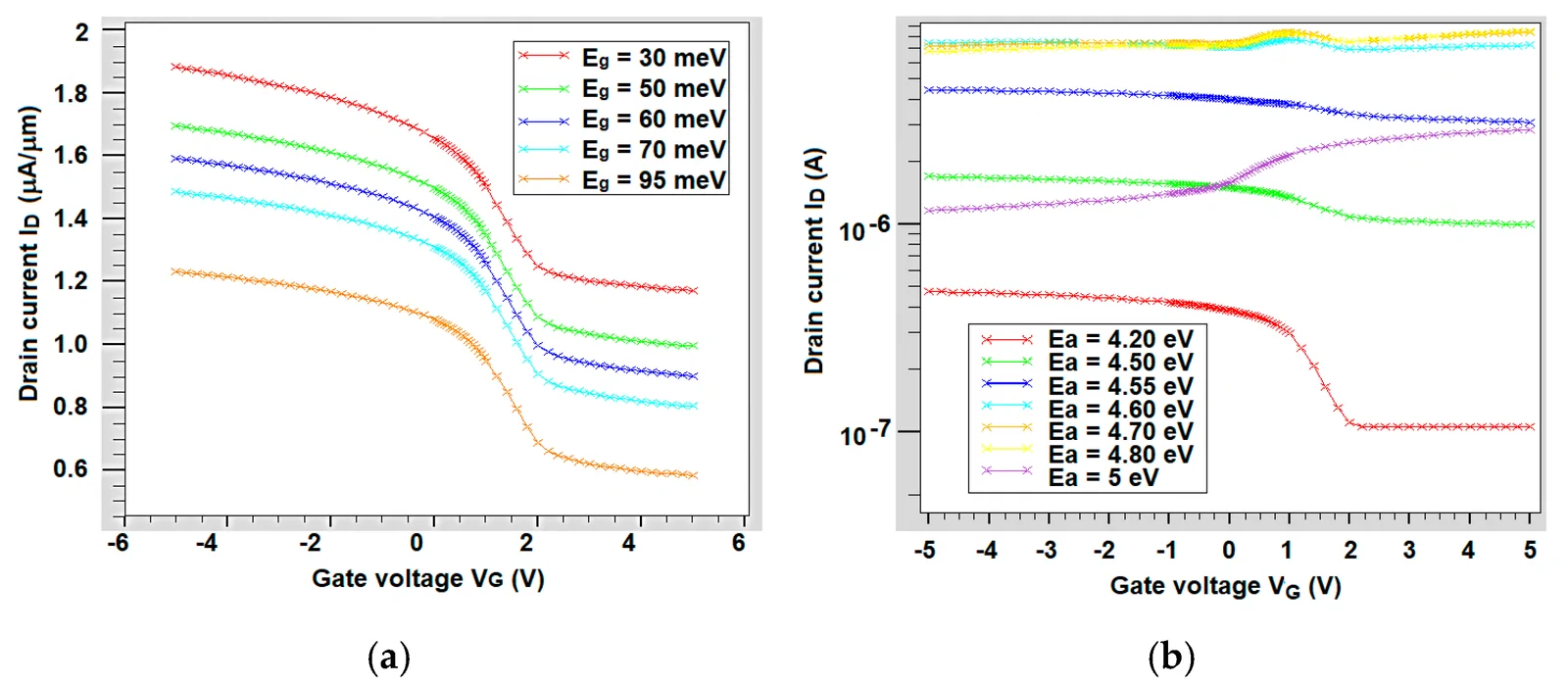
Different transfer characteristics simulated at V_DS_ = +2 V for GFET at different: (**a**) E_g_ values at linear scale; (**b**) Ea values at log scale.

Due to the lack of graphene material in the library, carbon-based semiconductor, like diamond, was considered in the first iteration. Preliminary simulations have shown that the use of high E_g_ values, E_g_ of 5.2 eV for diamond or 1.1 eV for silicon or 0.67 eV for Ge, provides currents that are three to six orders of magnitude lower than the currents simulated at E_g_ = 60 meV [56]. These previous studies confirm that E_g_ must be sub-100 meV [56]. Thus, Figure 7a presents I_D_-V_G_ simulated characteristics, at V_DS_ = 2 V, for all parameters matching those of the first iteration, except for E_g_. As observed in this figure, decreasing E_g_ from 100 to 30 meV leads to an increase in the drain current, albeit by minor increments (e.g., from 1.2 μA/μm to 1.9 μA/μm).

At V_G_ = −5 V, the measured drain current is still much higher than simulated current for each of the two types of FET. In simulations, the adjusted value of E_g_ slowly reduces the error. Therefore, the optimal E_g_ value is chosen closer to the previous experiments [44,45,46], for GNW-FET, E_g_ = 50 meV, and, for NCG-FET, E_g_ = 70 meV.

A more interesting analysis results from the simulations made in Figure 7b. Here all parameters are maintained constant (including V_DS_ = 2 V and E_g_ = 50 meV); only the electron affinity in graphene, Ea, is adjusted, a parameter available in the “material” statement.

In the next discussions, the simulated current I_D,sim_ is monitored and compared to experimental current I_D,exp_ at the minimum voltage, V_G_ = −5 V, representing always the maximum current for all experimental GFETs; Figure 3. In Figure 7b, at V_G_ = −5 V, I_D,sim_ jumps from 1.6 μA/μm when Ea is 4.5 eV to 7 μA/μm when Ea is 4.6 eV. Forcing Ea above 4.7 eV no longer increases the current and all the simulated currents are saturated sub-7 μA/μm. Also, for Ea = 4.8 eV … 5 eV, the conduction type in graphene is reversed: from ambipolar conduction with maximum holes current, as in the experiment, Figure 3, to ambipolar conduction with maximum electron current, which is non-useful for our GFETs.

**Figure 8:**
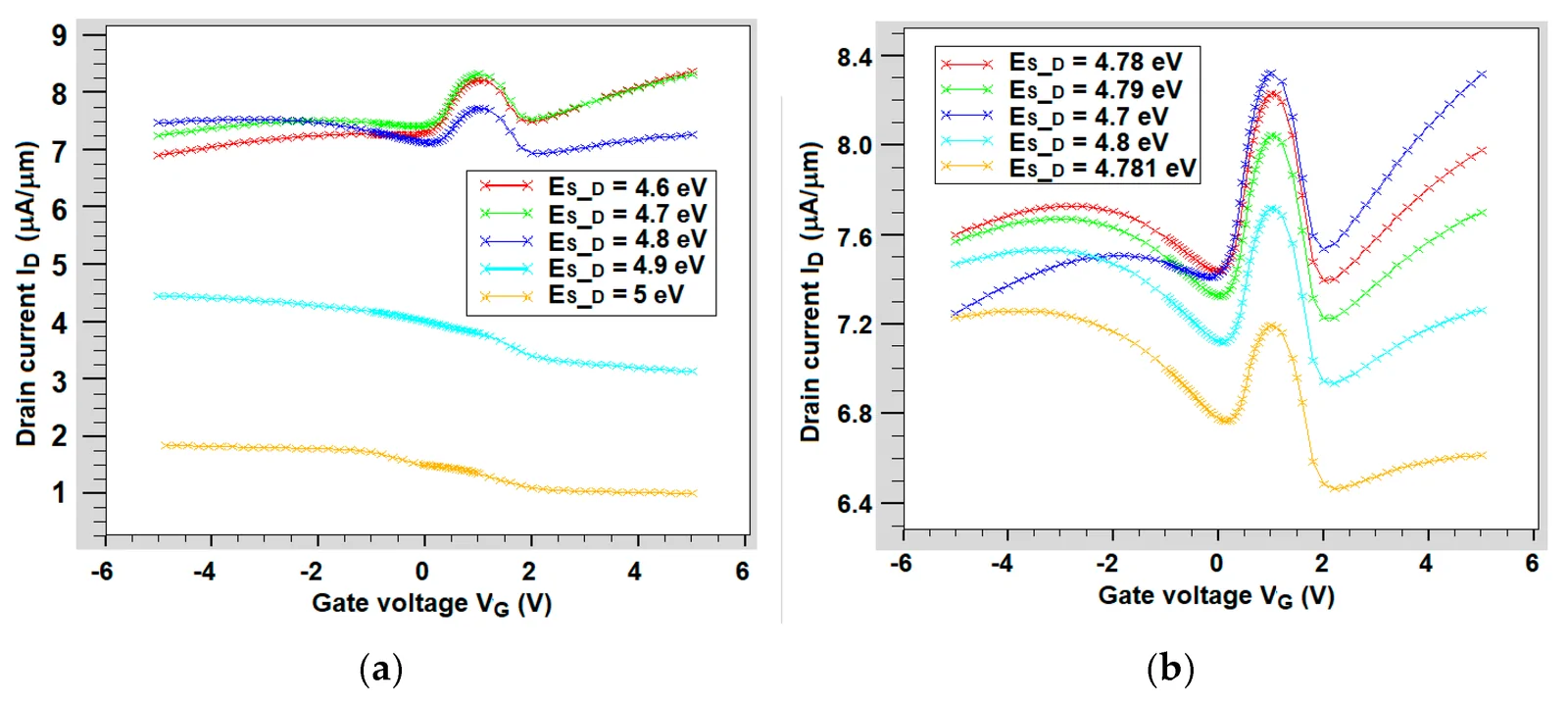
Different transfer characteristics of GFET, simulated at V_DS_ = +2 V at different E_S_D_ values: (**a**) coarse tuning; (**b**) fine tuning.

Conversely, reducing Ea below 4.5 eV, towards 4 eV leads to massive current decrease, a useless direction for calibration; Figure 3. Consequently, an optimal value Ea = 4.6 eV is selected for the generic GFET, which also reveals an incipient sketched Dirac point in simulations from Figure 7b that becomes more pronounced in Figure 8a.

It is important to match all energy values. If Ea level is inappropriate to E_S_D_ level, then source/drain contacts can be converted from ohmic to Schottky contacts, which prevent the current flow, a phenomenon frequently encountered in transistors with ambipolar conduction [14,58,59,60,61]. In a reference experiment [45], this issue was successfully mitigated utilizing gold contacts for source, drain and gate metals.

Therefore, the third energetic parameter, which offers optimization of the contact resistances, is the metal work function for the source/drain contacts, E_S_D_, available in the “contact” statement. Although the GFETs were ideally fabricated with Au contact, a 20 nm Cr layer was used for adherence This bimetallic combination, combined to inherent impurities existent during the experimental deposition process, may lead to variations in E_S_D_. The next figure comparatively presents the simulated I_D_-V_G_ characteristics at V_DS_ = 2 V, with all parameters from the I-st iteration, using updated values E_g_ =50 meV and Ea = 4.6 eV, while varying E_S_D_. This optimization is performed first via coarse tuning from 5 eV for pure Au to 4.6 eV for pure Cr, Figure 8a, then by fine tuning around 4.7 eV value, Figure 8b.

Whether E_S_D_ is either increased above 4.82 eV or decreased below 4.77 eV, the current decreases compared to the experimental value and the simulations are no longer relevant. At V_G_ < 0, the maximum hole current, I_D,sim_ = 7.7 μA/μm (Figure 8b, red curve), occurs if E_S_D_ = 4.78 eV, which is still lower than I_D,exp_ in GNW and NCG in Figure 3.

Finally, to this II-nd iteration, for each type of graphene material, the optimal parameters were selected independently by minimizing the simulation/measurement Er − g function, given by Equation (4), at V_DS_ = 2 V.

The complete set of GFET parameters, hereinafter referred to as *Set-II*, contains the *Set-I* parameters, plus the following updated and calibrated parameters:

- For GNW-FET, the updated parameters are: E_g_ = 50 meV, Ea = 4.6 eV, and E_S_D_ = 4.8 eV, which match the experimental values, reducing the global error at log scale for current, and Er − g from 43.41% for Set-I to 25.22% for Set-II, according to Equation (4).

- For NCG-FET, the updated optimal parameters are: E_g_ = 70 meV, Ea = 4.61 eV, and E_S_D_ = 4.79 eV, which reduces the global error at log scale for current, and Er − g from 112.07% for Set-I to 67.52% for Set-II, according to Equation (4).

### 3.3. GFET Simulation at the III-rd Iteration—Charge Carriers Modulation Consideration

In the III-rd iteration, those parameters that are related to the properties of charge carriers in graphene are investigated. For both the GNW and NCG materials, the last simulated drain currents are still lower than those obtained from experimental GFETs [45,46] or currents in silicon FETs [48,50]. This fact is inconsistent with 10 times higher mobility in graphene versus silicon. Until now, only the CVT model of carrier’s mobility was used. This model is especially recommended for unipolar transistors working with inversion channels, like MOSFETs [40,47,60,61], insisting on a strong mobility attenuation with interfaces, temperature, electric field and impurities.

After simulating Set-I and Set-II with the CVT mobility model, as a standard benchmark, a systematic lack of current sensitivity to the mobility values was systematically noticed. This is because, even if μ_n_ and μ_p_ were varied by thousands, up to 15,000 cm^2^/Vs, the CVT model always attenuates mobility to around 800 cm^2^/Vs for μ_n_ and 400 cm^2^/Vs for μ_p_, as in traditional semiconductors, a case that is not appropriate in GFETs.

A more suitable model for two-carrier conduction is Klaassen (KLA) model [62], which is a specific model to bipolar conduction [40], but still suffering from severe attenuation of the carriers’ mobility. Some textbooks recommend CONMOB [40] or ARORA [63] as appropriate mobility models for low electric fields, but only CONMOB is also suitable for un-doped semiconductors, such as our graphene, for which the mobility attenuation with impurities is unnecessary. However, CONMOB model is not a constant 100% mobility model; it still takes into account the mobility attenuation with the lattice temperature [40].

**Figure 9:**
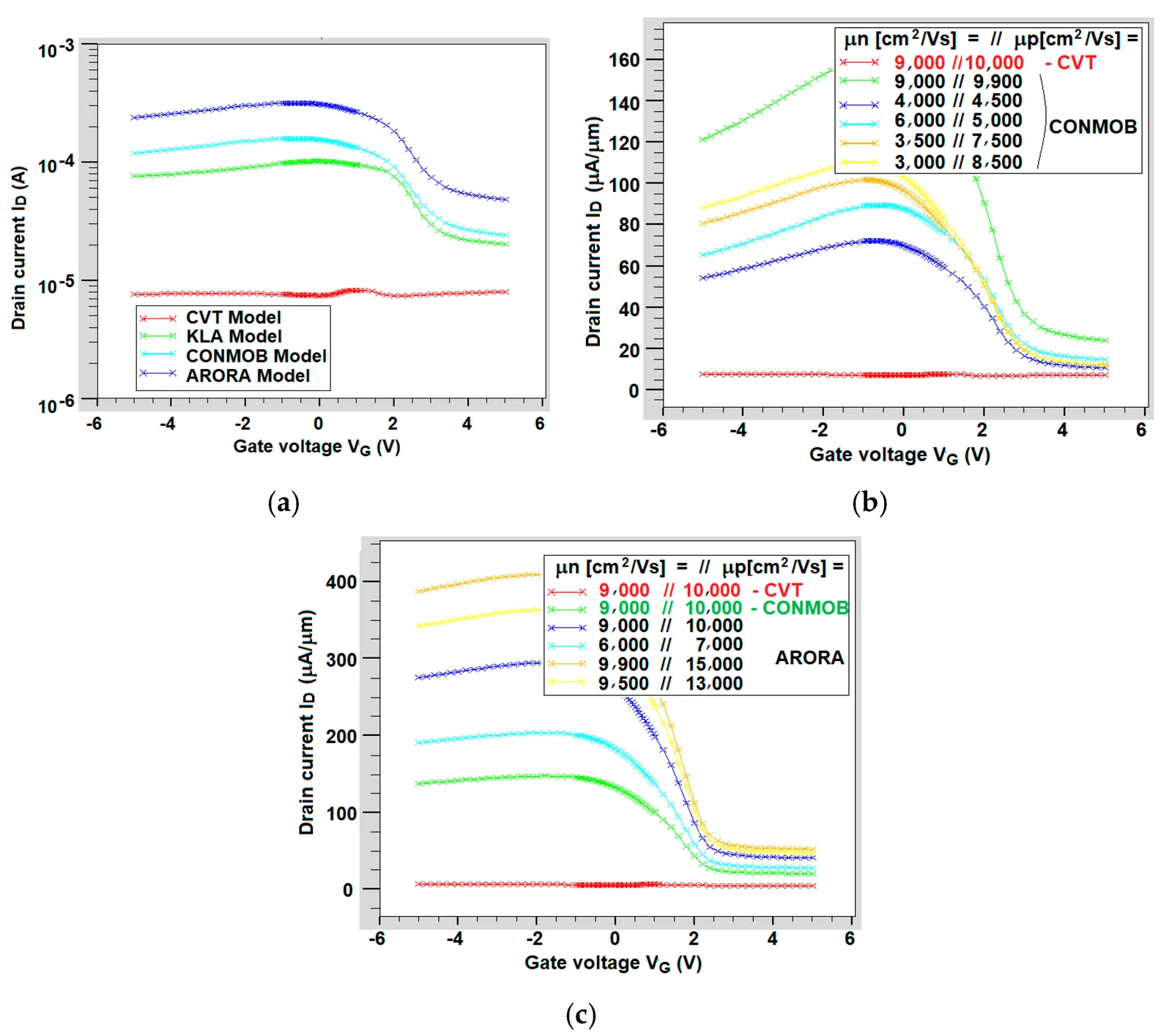
Different transfer characteristics simulated at V_DS_ = +2 V for: (**a**) generic GFET keeping constant the mobility; (**b**) GNW device selecting CONMOB model and changing the mobility values; (**c**) NCG device selecting Arora model and changing the mobility.

Figure 9a shows the transfer characteristics of a generic simulated GFET for these four models, keeping all the Set-II parameters constant and explicitly defining μ_n_ = 9000 cm^2^/Vs and μ_p_ = 10,000 cm^2^/Vs for all curves. Taking into account the proximity of the simulated curves in Figure 9a with the experimental ones in Figure 3, the following mobility models are selected as optimal in the next simulations:

- CVT model is not appropriate for GFETs;

- KLA model can be sometimes appropriate for low currents that flow through ultra-thin graphene film, like SLG, where strong mobility attenuation can arise;

- CONMOB model is appropriate for GNW-FET;

- Arora model is appropriate for NCG-FET.

The results from Figure 9a enable a comparative study of the transfer characteristics for both GFETs, the Set-II parameters except mobility, empirically selecting their values in the “mobility” statement, in the vicinity of the theoretical values [40].

Based on Figure 9b,c analysis, *Set-II* parameters are updated to establish Set-III, incorporating the optimal mobility models and values: (i) for GNW: CONMOB model, μ_n_ = 9000, and μ_p_ = 9900 [cm^2^/Vs]; (ii) for NCG: Arora model, μ_n_ = 9500, and μ_p_ = 13,000 [cm^2^/Vs]. By implementing these adjustments, a close agreement with the experimental current envelope is achieved for both transistors.

The calibration continues by using *Set-III* parameters, manipulating now the electrons/holes concentration, via Nc, N_V_ values, available in the “material” statement.

**Figure 10:**
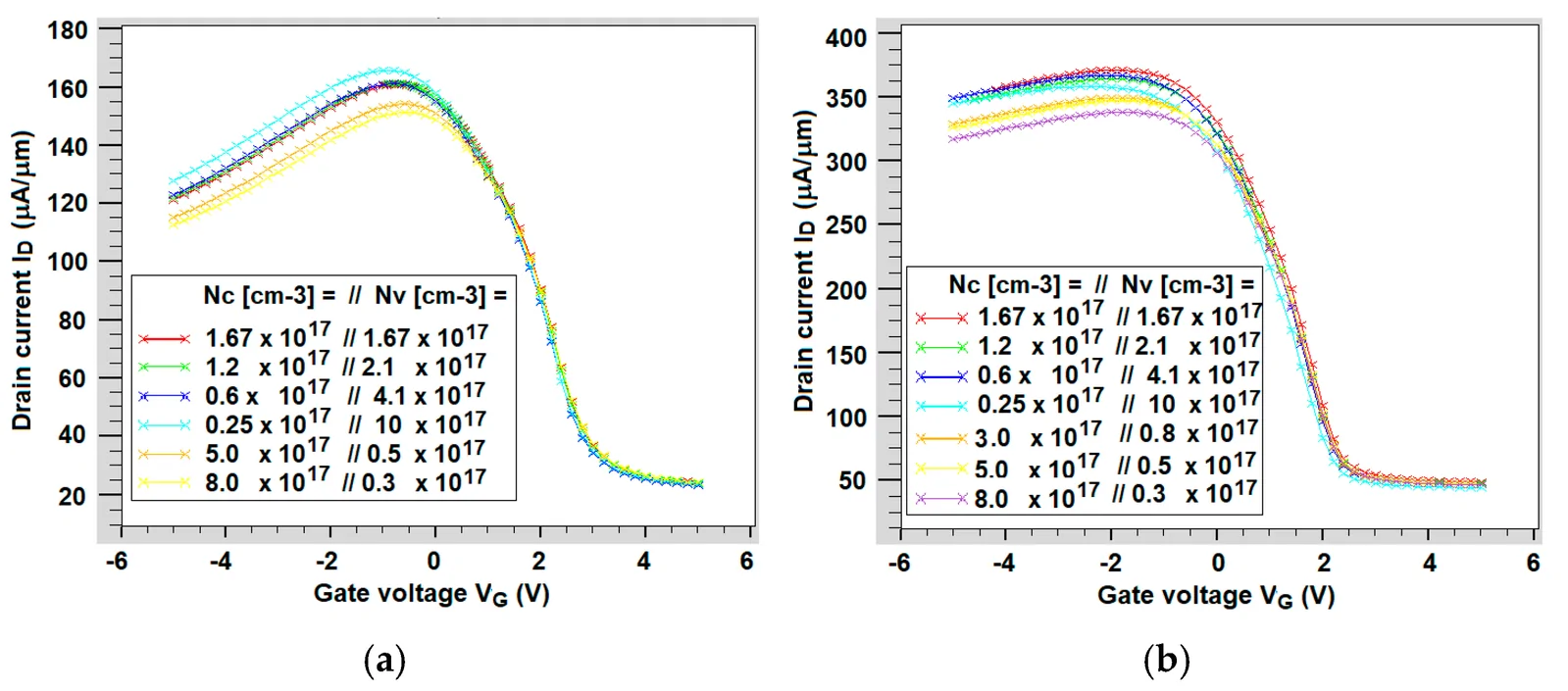
Different transfer characteristics simulated at V_DS_ = +2 V, at linear scale, for different Nc, N_V_ values for: (**a**) GNW-FET; (**b**) NCG-FET.

The density of states must fulfill: N_V_/N_C_ ranging around unity, in order to take into account the lattice symmetry, the electron/hole effective mass, and the experimental currents from Figure 3, which means: |I_D_| _VG<0_ > |I_D_| _VG>0_. Figure 10a,b shows the transfer characteristics at linear scale of the simulated GNW-FET and NCG-FET devices, keeping the *Set-III* parameters and changing N_C_ and N_V_. If the previous constraints are applied, only small deviations of the I_D_-V_G_ curves are observed, and the optimal set—N_C_, N_V_—is chosen in accordance with the minimization of the error function Er − g. It is possible that the intrinsic carrier concentration, n_i_, differs from one synthesized graphene material to another. Consequently, n_i_ could deviate from standard literature estimations, such as 5 × 10^16^ cm^−3^. According to Equation (1) a wider spectrum of values for the N_C_, N_V_ set could result, but this specific case lies outside the scope of the present study.

At the end of this III-rd iteration, following the minimization of Er − g function, the final list of *Set-III* parameters is updated by the next optimal values:

For GNW-FET, the updated parameters are: CONMOB mobility model, μ_n_ = 9000 cm^2^/Vs, μ_p_ = 9900 cm^2^/Vs, N_C_ = 1.6 × 10^17^ cm^−3^, N_V_ = 1.6 × 10^17^ cm^−3^, reducing the global error for current, and Er − g from 25.22% for *Set-II* to 7.63% for *Set-III*, according to Equation (4).

For NCG-FET, the updated optimal parameters are: Arora model, μ_n_ = 9500 cm^2^/Vs, μ_p_ = 13,000 cm^2^/Vs, N_C_ = 1.2 × 10^17^ cm^−3^, N_V_ = 2.1 × 10^17^ cm^−3^, reducing the global error for current, and Er − g from 67.52% for *Set-II* to 14% for *Set-III*, according to Equation (4).

For a generic GFET, CONMOB model seems to be convenient, offering more flexibility in the adjusting of the drain current. The reunion of these final parameters forms the actual *Set-III* parameters for GFETs.

### 3.4. GFET Simulation at the IV-th Iteration—Defects Consideration

**Figure 11:**
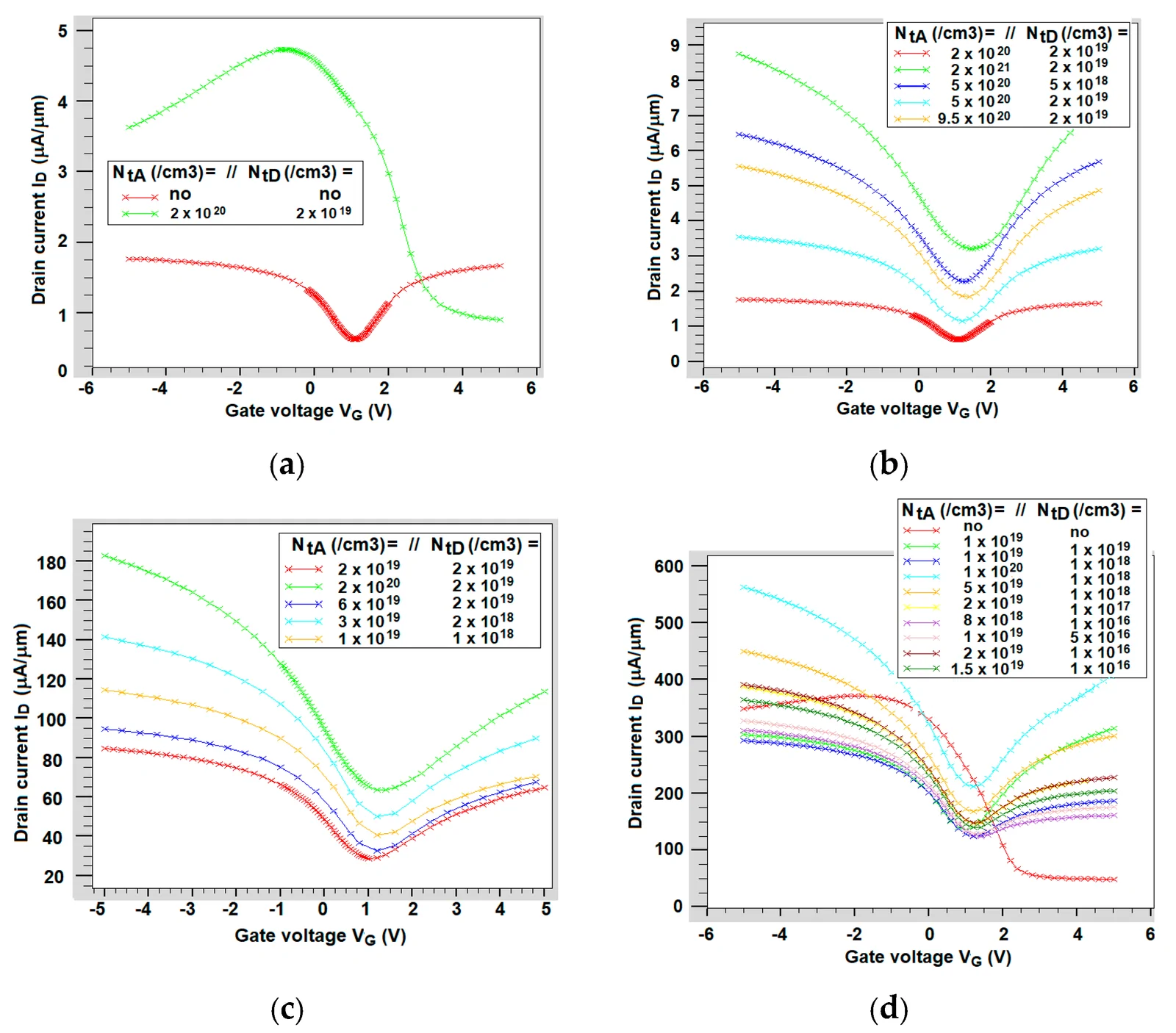
Different transfer characteristics simulated at V_DS_ = +2 V for different N_tA_, N_tD_ values for: (**a**) a generic GFET with and without defects; (**b**) a generic GFET with multiple N_tA_ and N_tD_ selections; (**c**) GNW-FET with multiple selections; (**d**) NCG-FET with multiple selections. The optimal defect levels, N_tA_, N_tD,_ are calibrated in Figure 11c,d to minimize the global error Er − g versus the experimental values from Figure 3.

Usually, at the graphene–insulator interfaces, some defects arise. As GFETs are representatives of the larger class of Thin-Film Transistors (TFT), their defects are simulated by the densities of acceptor and donor traps, N_tA_ and N_tD_, while their capture cross-sections for electrons and holes, SIGMAE and SIGMAH, are maintained constant to their default values [40]. In some TFTs, the existence of these defects represents the primary conduction mechanism [64,65]. Hence, the performance of our GFETs is expected to be highly dependent on N_tA_ and N_tD_ as well [45]. Figure 11a shows the transfer characteristics of the simulated generic GFET with the defects statement both activated and disactivated. If the defects are taken into account, the Dirac point clearly emerges as a minimum on the simulated characteristics; Figure 11a. This simulation, by successfully resolving the Dirac minimum on the I_D_-V_G_ curve, has just accomplished the second aim of this paper [45,52,57,61]. In the next figures, different values of N_tA_, N_tD_ are selected by trial, keeping constant the *Set-III* parameters, for a generic GFET device. The simulated curve presents a Dirac minimum in the case of defects level around N_tA_ = 2 × 10^20^ cm^−3^ and N_tD_ = 2 × 10^19^ cm^−3^ versus the case without defects; Figure 11a. In Figure 11b it is observed that quite different values of N_tA_ and N_tD_, considered by attempt, do not lead to monotonic increases in current. They are simulated because they can randomly and uncontrollably occur in the laboratory practice. It is also observed that particular values of these two defect densities can lead to an accentuation of the Dirac minimum (e.g., N_tA_ = 2 × 10^21^ cm^−3^ and N_tD_ = 2 × 10^19^ cm^−3^; Figure 11b). Figure 11c,d present the I_D_-V_G_ simulated curves for different N_tA_ and N_tD_ pairs for GNW-FET and NCG-FET, respectively.

**Figure 12:**
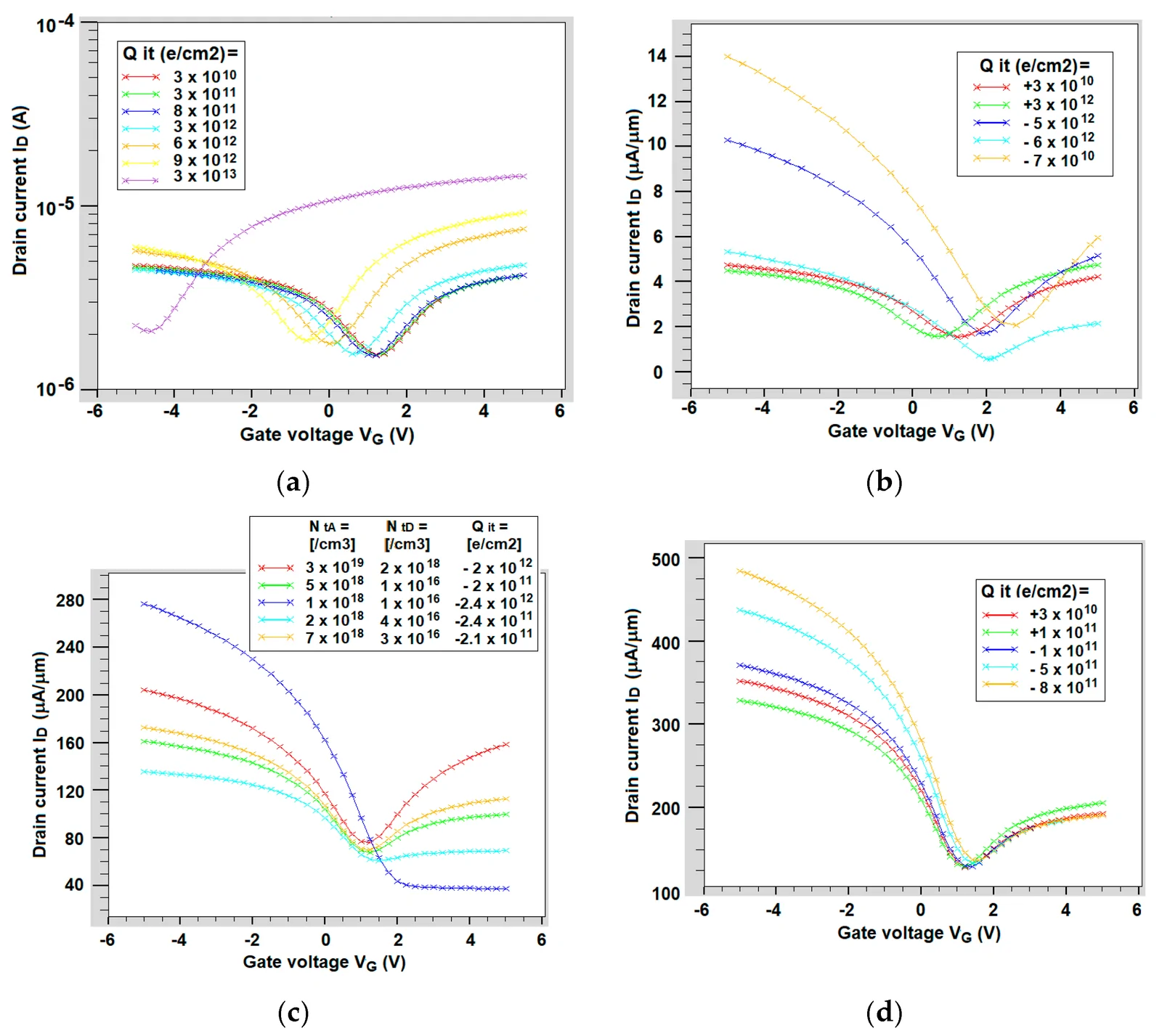
Different transfer characteristics simulated at V_DS_ = +2 V for different Q_it_: (**a**) positive Q_it_ shifts the I_D_-V_G_ curve for a generic GFET; (**b**) negative Q_it_ shifts the I_D_-V_G_ curve for a generic GFET; (**c**) N_tA_, N_tD_, Q_it_ recalibrations for GNW-FET for Set-IV; (**d**) Q_it_ calibrations for NCG-FET, for Set-IV, keeping N_tA_ = 1.5 × 10^19^ cm^−3^, N_tD_ = 10^16^ cm^−3^.

The last analysis aims to resolve the remaining mismatch between the experimental and simulated characteristics at V_DS_ = 2 V; in the experiment the Dirac current shifts to higher voltages than V_G_ = 1 V, while in the simulations it appears around V_G_ = 1 V. In this scope, the parameter responsible for the horizontal shift of the I_D_-V_G_ curve is modeled via Q_it_ interface charge as the interface flat-band charge [47,51,53], given in electrons charge per cm^2^ at the interface of the graphene film with insulators; Figure 12.

The interface charge Q_it_ was extensively studied in MOSFETs while it is typically represented by positive electric charges that contaminate the oxide, like Na+, K+, or positive clusters [SiO_x_]^+^ [61,65]. We suspected that the measured GFET suffered from contaminations too. Firstly, their levels are considered to be in a range from a minimal value of Q_it_ = +3 × 10^10^ e/cm^2^, to +3 × 10^11^, up to a maximum of +3 × 10^13^ e/cm^2^ for a generic GFET; Figure 12a,b. Figure 12a proves the I_D_-V_G_ leftward shift, with an increasing positive value of Q_it_. But our experimental Dirac points are rightward shifted versus V_G_ = 1 V. This deviation requires negative values for Q_it_; Figure 12b. When Q_it_ < 0, the rightward shift of the Dirac peak occurs at voltages exceeding V_G_ = 1 V, in excellent agreement with experimental data in Figure 3. Further analyses in Figure 12c,d present the simulated variations in Q_it_, tailored for the calibration of the GNW-FET and NCG-FET, respectively. It is observed that a negative Q_it_ charge is responsible for the current increasing, because the negative interface charge stimulates the formation of hole accumulation channels.

Potential candidates that explain the presence of the negative Q_it_ charge could be adsorbed O_2_/H_2_O in any ionic state or polymer residues commonly found in processed graphene [66] for both devices.

The optimization of the simulated curves relative to the experimental data points was performed without altering the previously established parameters from Set-I, II and III, following the supreme goal—the error function minimization. Sometimes, some high negative values for Q_it_ increase the drain current so much, so that N_tA_ and N_tD_ parameters were re-adjusted for GNW-FET; Figure 12c. For NCG-FET, the optimal value for Q_it_ was searched while N_tA_ = 1.5 × 10^19^ cm^−3^ and N_tD_ = 10^16^ cm^−3^; Figure 12d. At the end of this IV-th iteration, the final list of parameters, as *Set-IV*, is updated by the following new values:

- For GNW-FET, the updated optimal parameters are: N_tA_ = 3 × 10^18^ cm^−3^, N_tD_ = 2 × 10^16^ cm^−3^, and Q_it_ = −2.2 × 10^11^ e/cm^2^, so that the global error decreases from Er − g = 7.63% for *Set-III* to 6.1% for *Set-IV.*

- For NCG-FET, the updated optimal parameters are: N_tA_ = 1.5 × 10^19^ cm^−3^, N_tD_ = 10^16^ cm^−3^, and Q_it_ = −1 × 10^11^ e/cm^2^, so that the global error decreases from Er − g = 13.93% for *Set-III* to 11.8% for *Set-IV.* The reunion of these final parameters forms the actual *Set-IV* parameters for all GFETs.

## 4. Discussions

Table 1 compiles the final parameter values, Er − g errors from both the first and final iteration, along with the RMSE values calculated for the last dataset, across all simulated GFETs. All these parameters were calibrated by minimizing the Er − g error function between the simulated and experimental curves at the median V_DS_ value, V_DS_ = +2 V, for each of the experimentally measured transistors: generic GFET, GNW-FET, and NCG-FET. Finally, for a generic GFET the optimal parameters were selected to encompass the widest possible current range, whereas the calibrations for the GNW-FET and NCG-FET directly targeted the specific experimental curves presented in Figure 3.

**Figure 13:**
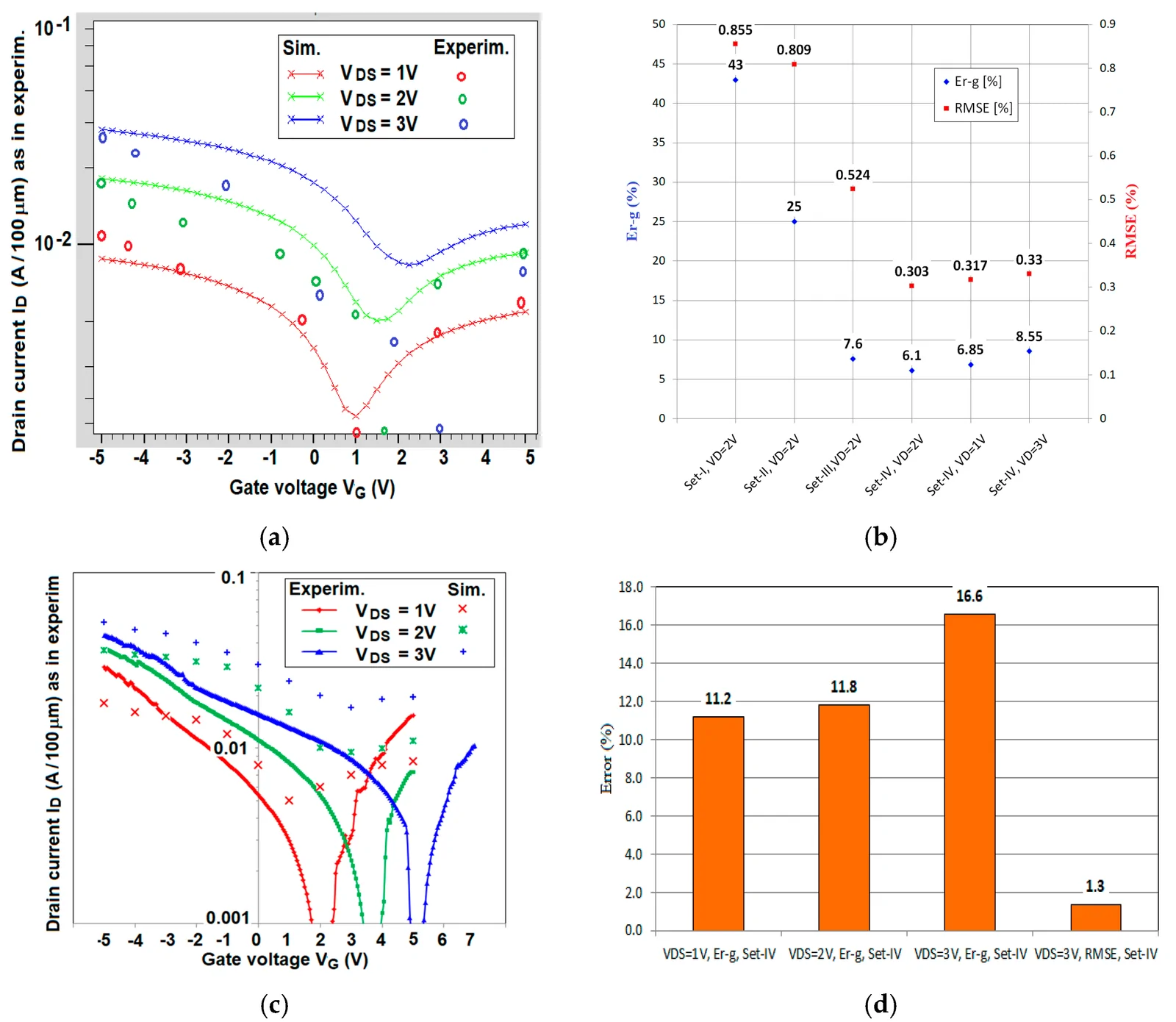
(**a**) Simulation versus experimental transfer characteristics for GNW-FET at V_DS_ = 1 V, V_DS_ = 2 V and V_DS_ = 3 V; (**b**) error evaluation for GNW-FET using Er − g and RMSE functions; (**c**) simulation versus experimental transfer characteristics evolution for NCG-FET at V_DS_ = 1 V, V_DS_ = 2 V and V_DS_ = 3 V; (**d**) global error and RMSE evolution for NCG-FET. The same width of the channel of 100 μm was considered for all simulated and measured transistors in this comparative figure.

Figure 13 comparatively presents the I_D_-V_G_ curves of the calibrated GFET transistors across the optimization stages, starting from Set-I to Set-IV, where the current is expressed in Ampere per 100 μm graphene film width. Obviously, a point of interest is an entire simulated family of transfer characteristics at all three drain-source voltages, V_DS_ = 1 V, V_DS_ = 2 V and V_DS_ = 3 V, as in experimental tests; Figure 3.

In Figure 13a, such a family of transfer characteristics is exemplified for GNW-FET; experimental picked points are added near the simulated curves. The global errors Er − g = 6.1 % but still suffer from a weak decrease in the simulated current, compared to the experimental current, around the Dirac point.

Figure 13b illustrates both the global error evolution, Er − g, and the RMSE, starting from the I-st Set to the last IV-th Set, for a visual estimation of the error decreasing for each V_DS_ applied voltage to GNW-FET. Similarly, Figure 13c presents the simulated points added near the experimental curves, also at V_DS_ = 1 V, V_DS_ = 2 V and V_DS_ = 3 V, for NCG-FET. Figure 13d presents the global error evolution, Er − g, for Set-IV, for each V_DS_ voltage applied to NCG-FET, and RMSE error at Set-IV and V_DS_ = 3 V for the same device. In general, the RMSE value is lower than Er − g, because the difference in the squares of two individual currents, for example, at V_G_ = −2 V and V_DS_ = 3 V, yields a value of (0.0183A − 0.035A)^2^ × 100 [%] = 0.0271 %, while Er − i = ||lg0.0183| − |lg0.035|/lg0.035| × 100 [%] = 16.2%.

From Figure 13a it is observed how the simulated curve fits the experimental one for GNW-FET at V_DS_ = 1 V and 3 V, for Set-IV, without any re-tuning. The global error Er − g remains slightly higher than for the optimized Set-IV at V_DS_ = 2 V but keeps the order of magnitude, being 6.85% at 1 V and 8.55% at 3 V. Choosing RMSE as the error function, the error moved around 0.3%, compared to the optimum at 2 V that offered 0.303%.

**Figure 14:**
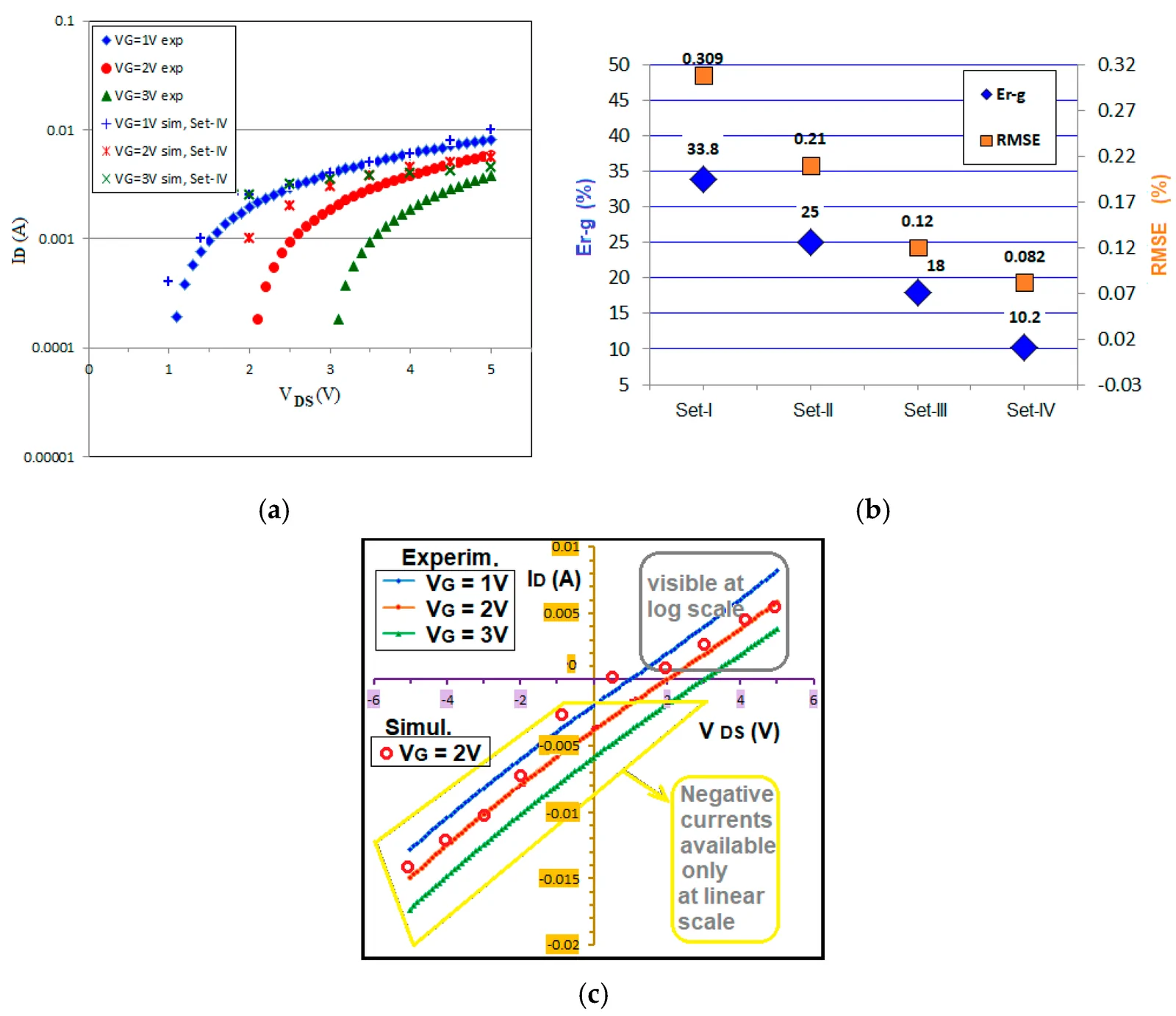
(**a**) Simulation versus experimental output characteristics for GNW-FET at V_G_ = 1 V, V_G_ = 2 V and V_G_ = 3 V, truncated only for positive currents; (**b**) estimated errors for GNW-FET at V_G_ = 2 V; (**c**) complete experimental output characteristics for GNW-FET, at linear scale, for −5 V < V_DS_ < +5 V, without negative drain current exclusion, and few simulated points for GNW-FET with Set-IV, only at V_G_ = 2 V (Simul.), to avoid any crowding.

Also, it is interesting to compare the output characteristics, I_D_-V_DS_, for at least one of the devices. In Figure 14a, the simulated points are superimposed onto the experimental I_D_-V_DS_ points at V_G_ = 1 V, 2 V and 3 V for GNW-FET. The errors are estimated in Figure 14b; Er − g decreases from 33.8 % to 10.2%, while RMSE decreases from 0.309 % to 0.082 %, from Set-I to Set-IV, which confirms the calibration method. However, I_D_-V_DS_ curves present high deviations at higher gate voltages.

Although the experimental I_D_-V_DS_ characteristics in Figure 14a appear unusual, since the curve for V_G_ = 1 V is plotted only for V_DS_ > 1 V, whereas the curve for V_G_ = 3 V is plotted only for V_DS_ > 3 V, it should be noted that this operation resulted from a simple truncation of the complete I_D_-V_DS_ curves measured within the −5 V < V_DS_ < +5 V range; Figure 14c. When a logarithmic scale was applied to the current, the software automatically excluded all negative I_D_ values. Figure 14c displays the complete experimental I_D_-V_DS_ curves on a linear scale, alongside few simulated I_D_-V_DS_ data points. Only the grey boxed region of the characteristics, at I_D_ > 0 from Figure 14c, is plotted on a logarithmic scale in Figure 14a. The purpose of the logarithmic representation was to visualize the drain current transition from a linear shape at low V_DS_ to a saturated current at high V_DS_ voltage, as shown in Figure 14a and similarly appearing in the experimental data from [45].

**Figure 15:**
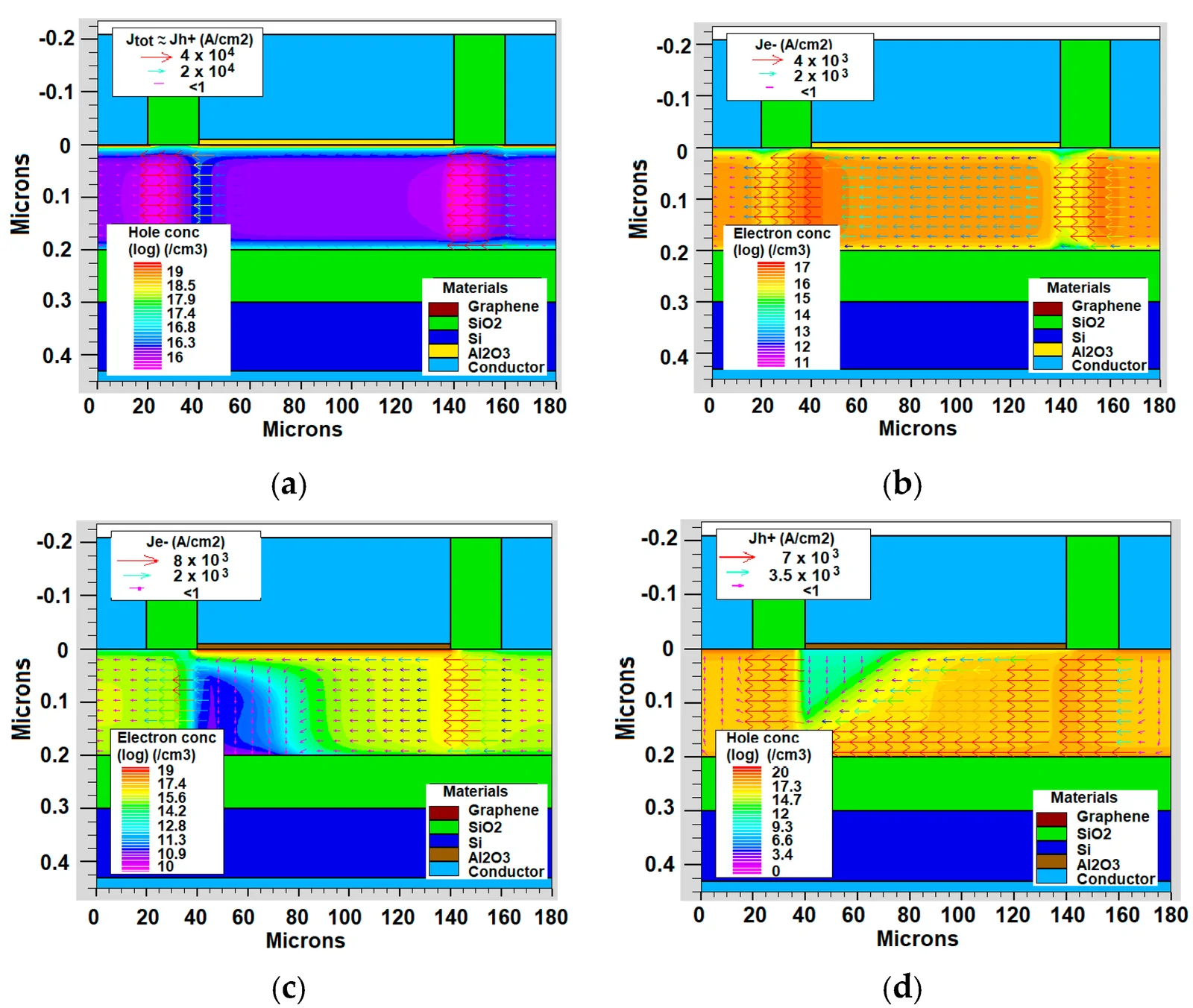
The simulated carriers distribution and their currents vector inside NCG-FET, biased to: (**a**) V_G_ = −5 V, V_SD_ = 2 V having holes accumulation at the superior interface; (**b**) V_G_ = −5 V, V_SD_ = 2 V having electrons pushed to the volume; (**c**) V_G_ = +5 V, V_SD_ = 2 V having electrons accumulation at the superior interface; (**d**) V_G_ = +5 V, V_SD_ = 2 V having holes pushed to the volume.

Another interesting analysis is to repeat the simulation of the ambipolar current flow, initiated in Figure 5, but now using the optimized data Set-IV. It is only checked for one representative, as NCG-FET, with the strongest current. Figure 15 presents the electrons and holes distribution inside the graphene NCG film and their current flow vectors at the extreme voltages, V_G_ = −5 V and V_G_ = +5 V, while V_DS_ = 2 V, to observe the carriers transport at interfaces and through the graphene bulk.

Compared to the preliminary simulations from Set-I in Figure 5, significantly higher values of current densities are now observed in Figure 15a–d, aligning much closer with the experimental data in Figure 3, due to the Er − g error decreasing. It is interesting to remark in all these cases that the gate systematically induces accumulation channels, normally at superior interface but also at inferior interface, very probably due to the defects consideration in simulations and in reality, acting as attractive ions or carrier traps.

The gate voltage plays another specific role in the graphene layer by spatially separating positive and negative carriers, so that their separate transport produces minimal recombination rates. This is a major difference between ambipolar and bipolar conduction.

**Figure 16:**
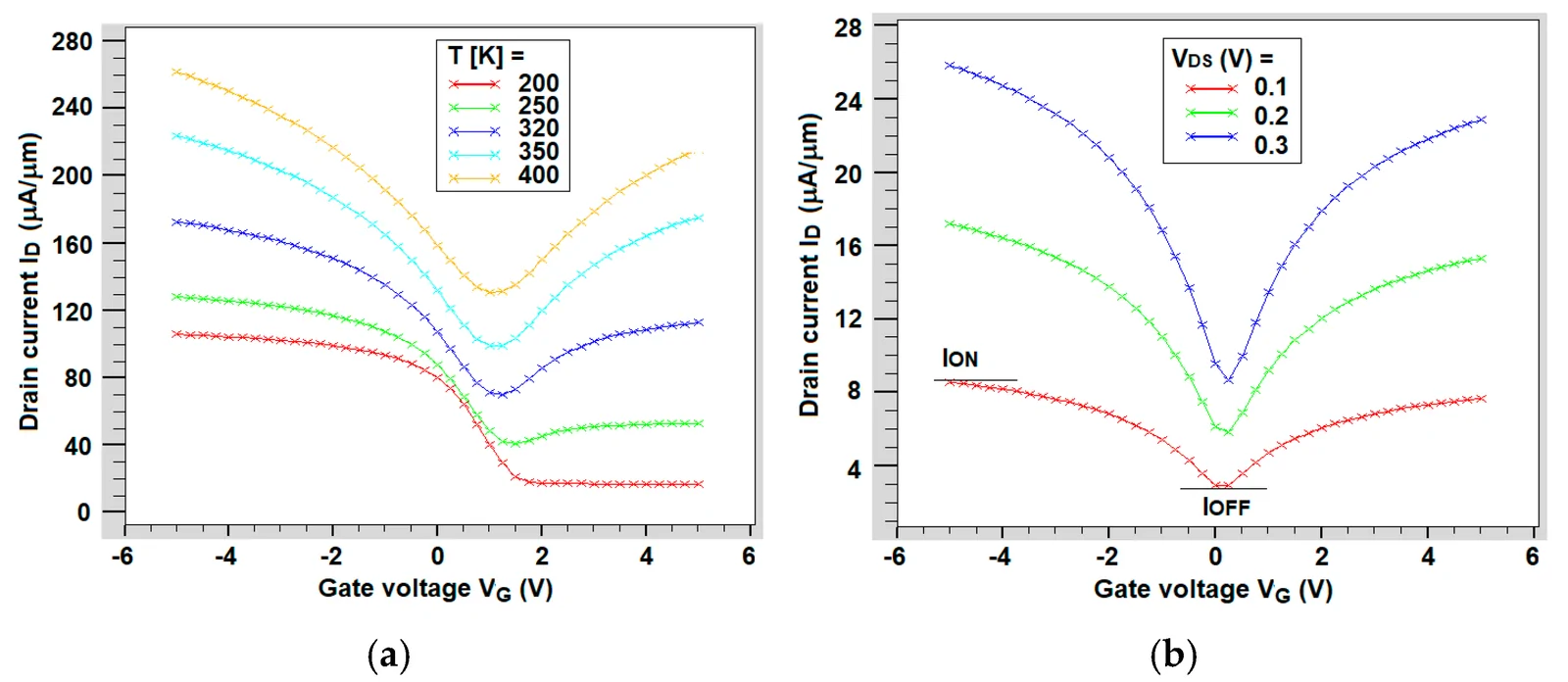
(**a**) The simulated I_D_-V_G_ curves of GNW-FET, I_D_-V_G_ at V_DS_ = 2 V, using Set-IV parameters for: (**a**) few temperatures; (**b**) low V_DS_ voltages at T = 300 K.

Another interesting discussion concerns a GFET simulation at a few distinct temperatures, following the I_D_-V_G_ evolution, using Set-IV parameters for GNW-FET device at V_DS_ = 2 V; Figure 16a. The results briefly prove the temperature influence on the drain current, via parameters E_g_, N_C_, N_V_, and Ea that present different temperature dependences. The GFET simulations with temperature do not have a dual temperature measurement for comparison. The current increasing with temperature is theoretically expected, and the simulations only offer the order of magnitude of expectancy when temperature increases from 200 K to 400 K.

A last discussion concerns the simulated I_D_-V_G_ curve around the Dirac minimum that exhibits a flatter transition, compared to the sharp experimental curves. Other papers reported experimental flatter transitions with I_ON_/I_OFF_ ratio around 3 [50], like our simulations, for GFETs with similar aspect and with a shape of the I_D_-V_G_ curve closer to the actual simulations; Figure 13a. A few key physical mechanisms have already been revealed to govern the Dirac point minimum: the defects presence by parameters N_tA_, N_tD_ and the contact barrier modulation by parameters E_S_D_ reported to Ea. A further phenomenon responsible for this behavior can be spatial puddle charge fluctuations. As a brief demonstration, if the drain-source voltage is reduced sub-0.5 V, prior mechanisms are alleviated, and the Dirac point becomes sharper in simulations; Figure 16b.

These low drain voltages, V_DS_ < 0.5 V, are more appropriate in the field of biosensors with GFET transducers, where the linear operating regimes are of interest. For instance, other authors fabricated a urea biosensor with graphene film, which was polarized at V_DS_ = 0.1 V, adjusting V_G_ voltage between −0.6 V ÷ +0.6 V, generating an I_ON_/I_OFF_ ratio of only 1.5 [67], while an aptamer-based FET-like electrochemical biosensor was operated at V_G_ = 0.1 V ÷ 0.5 V, with a similar flat transition, generating an I_ON_/I_OFF_ ratio around 1.9, [68], flatter and lower than even the simulated curves from Figure 16b that are around 2 ÷ 3.

## 5. Conclusions

This paper was focused on the GFET simulation as a device itself and not as a component from a circuit. First, a list of sensitive model parameters directly dependent on the graphene material properties was identified to enable precise control over the simulated drain current. Second, an empirical calibration methodology was presented, to align the GFET transfer characteristics achieved by simulations with the experimental recorded curves. The calibration method has the following main recommendations: (i) an initial selection of the material energy parameters (e.g., E_g_, Ea, and E_S_D_) is necessary to simulate a particular synthesized graphene, with defined semiconductor or quasi-metal properties, such as GNW or NCG; (ii) a subsequent selection envisages the selection of those parameters that characterize the charge carriers (e.g., mobility, convenient models for mobility, and densities of energetic states Nc, Nv) to scale the baseline level of the simulated current to the measured values; (iii) a final selection concerns the parameters for defects and traps (e.g., N_tA_, N_tD_, and Q_it_), which play a decisive role in the correct conduction of TFT transistors and, in particular, GFETs, these being the cornerstone that generated characteristics with Dirac minima and its lateral translation, matching experimental observations. The parameters selection was empirically done, by trials, targeting the minimization of an error function. Moving away from the conventional CVT model in favor of frameworks that do not severely degrade carrier mobility due to impurity concentration or inversion channel phenomena represented a key step forward for measurement-experiment optimizations. For instance, CONMOB or Arora model is able to provide simulated mobility in the range of thousands or tens of thousands of cm^2^/Vs, as the literature indicates for carrier mobility in graphene.

This paper also carries a theoretical impact, as the simulations, accompanying the experimental measurements, highlight the difference between ambipolar conduction, with charges of opposite sign flying on different paths, and classical bipolar conduction based on the encountering and recombination of electrons and holes.

Therefore, the calibration methodology presented in this paper does not guarantee that it obtains the most optimal parameters in the end, nor that the set of extracted parameters is the unique set that minimizes the error function, but it rather offers directions for searching for some physical parameters in the realm of GFET simulation as a device and not as an element connected in a circuit.

## Figures and Tables

**Table 1:** The final values of parameters that minimize the error function.

GFET Type/ Parameter	Generic–GFET	GNW-FET	NCG-FET
E_g_ (meV)	60	50	70
Ea (eV)	4.605	4.6	4.61
E_S-D_ (eV)	4.795	4.8	4.79
N_C_ (cm^−3^)	10^17^ ÷ 10^18^	1.6 × 10^17^	1.2 × 10^17^
N_V_ (cm^−3^)	10^18^ ÷ 10^17^	1.6 × 10^17^	2.1 × 10^17^
μ_n_-graphene (cm^2^/Vs)	~9300	9000	9500
μ_p_-graphene (cm^2^/Vs)	~11,000	9900	13,000
μ-model	CONMOB	CONMOB	ARORA
Q_it_ (e/cm^2^)	+10^12^ ÷ −10^13^	−2.2 × 10^11^	−1 × 10^11^
Acceptor traps N_tA_ (cm^−3^)	10^21^ ÷ 10^18^	3 × 10^18^	1.5 × 10^19^
Donor traps N_tD_ (cm^−3^)	10^19^ ÷ 10^16^	2 × 10^16^	1 × 10^16^
V_DS_ (V)	2	2	2
Initial Er − g at Set-I	-	43.7%	112.1%
Final Er − g at Set-IV	-	6.1%	11.8%
Final RMSE at Set-IV	-	0.303%	1.2%

## Data Availability

Data available in repository of Politehnica Bucuresti.

## Abbreviations

The following abbreviations are used in this manuscript:

FET	Field Effect Transistor
GFET	Graphene Field Effect Transistor
GNW-FET	Graphene Nano-Walls Field Effect Transistor
NCG-FET	Nano-Crystalline Graphene Field Effect Transistor
GTFET	Graphene Tunnel Field Effect Transistor
MOSFET	Metal Oxide Semiconductor Field Effect Transistor
SLG	Single-Layer Graphene
GNW	Graphene Nano-Walls
NCG	Nano-Crystalline Graphene
Er − i	Individual point error
Er − g	Error–global
V_G_	Gate Voltage
V_DS_	Drain-Source Voltage
RMSE	Root Mean Square Error
SEM	Scanning Electron Microscopy
TCAD	Technology Computer-Aided Design
TFT	Thin-Film Transistor
